# Spatial transcriptomic mapping of anti-MDA5 dermatomyositis reveals perivascular enrichment of type I interferon responses and immune activation

**DOI:** 10.1016/j.isci.2026.118072

**Published:** 2026-11-02

**Authors:** Yulong Qiao, Gexin Liu, Michael Tim-Yun Ong, Hao Sun, Ho So, Huating Wang

**Affiliations:** 1 Department of Orthopaedics and Traumatology, Li Ka Shing Institute of Health Sciences, The Chinese University of Hong Kong, Hong Kong SAR, China; 2 Center for Neuromusculoskeletal Restorative Medicine, Hong Kong Science Park, Hong Kong SAR, China; 3 Department of Bioinformatics, Warshel Institute for Computational Biology, School of Medicine, The Chinese University of Hong Kong, Shenzhen, Guangdong, China; 4 Department of Medicine and Therapeutics, Faculty of Medicine, The Chinese University of Hong Kong, Hong Kong SAR, China

**Keywords:** myositis, spatial transcriptomics, anti-MDA5 dermatomyositis, type I interferon, vasculopathy

## Abstract

Anti-MDA5 autoantibody-positive dermatomyositis (MDA5+ DM) is a rare autoimmune myopathy associated with rapidly progressive interstitial lung disease and a high mortality rate, yet its underlying molecular mechanisms remain poorly understood. To investigate this, we profiled the spatial transcriptome of muscle tissues from healthy controls, MDA5+, and MDA5- patients. We found that MDA5+ muscles exhibit a distinct spatial transcriptomic architecture driven by profound microenvironmental remodeling, accompanied by focal immune infiltration. Crucially, spatial niche analysis revealed that the type I interferon (IFN-I) signature is highly enriched within the endothelial cell (EC) niche. This perivascular niche also displayed enriched TGF-β, NF-κB, hypoxia, and TNF-α signaling, alongside transcriptomic signatures indicative of heightened inflammation and compromised oxygen transport. Altogether, our results characterize the distinct vascular pathology of MDA5+ DM muscles and provide a spatial mechanistic rationale for targeting these perivascular inflammatory pathways.

## Highlights

• Spatial transcriptomics maps muscle microenvironments in MDA5+ dermatomyositis
• IFN-I signatures are highly enriched within the perivascular niche of MDA5+ muscles
• Endothelial niches show elevated TGF-β, NF-κB, and TNF-α signaling in MDA5+ muscles
• Macrophage infiltrates associate with humoral immune responses in MDA5+ muscles

## Graphical abstract

**Figure:**
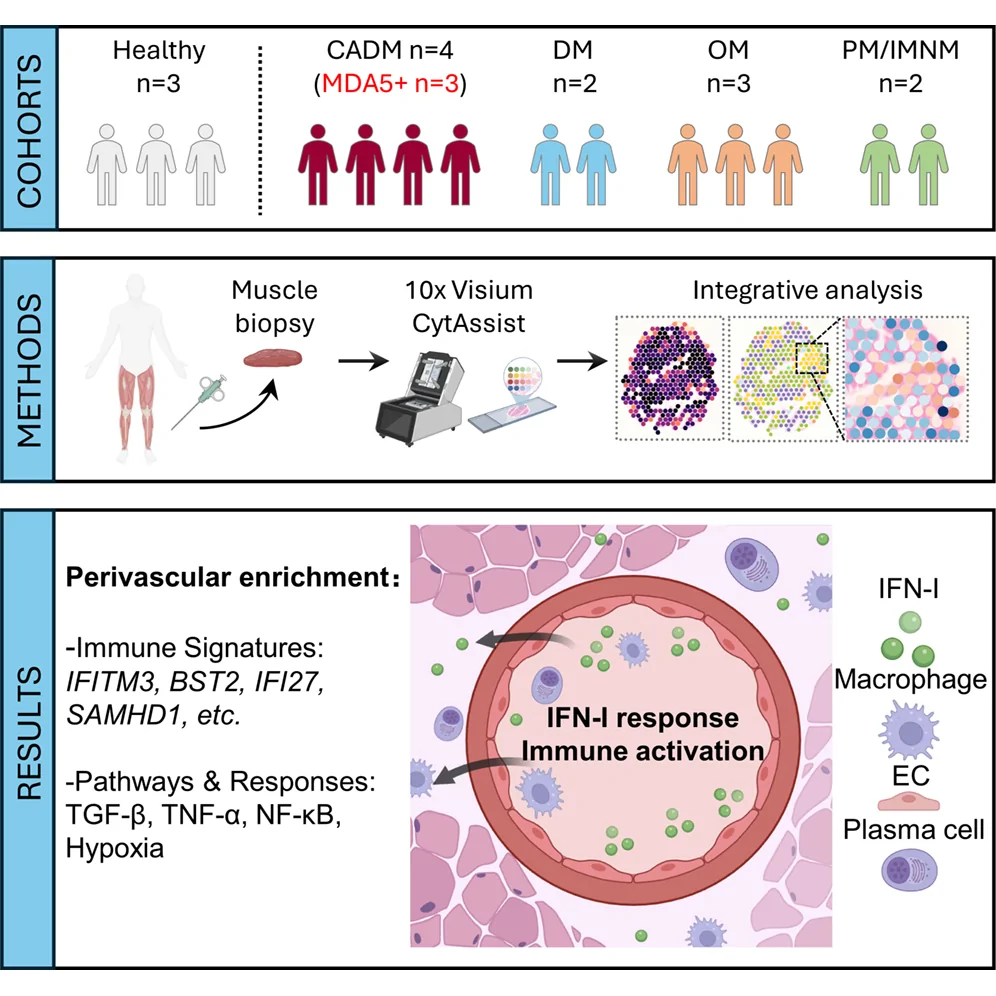

## Teaser

Biological sciences; Immunology; Immune system; Immune response; Components of the immune system; Bioinformatics

## Introduction

Idiopathic inflammatory myopathies (IIMs), also known as myositis, are a rare and heterogeneous group of autoimmune disorders characterized by immune-mediated muscle damage. In addition to muscles, other organs are frequently affected, such as skin, lungs, joints, and the gastrointestinal tract, leading to morbidity and even mortality.^1^ The discovery of myositis-specific autoantibodies (MSAs) improved the diagnosis and classification of patients into more homogeneous groups, outcome prediction, treatment strategies, as well as the understanding of molecular mechanisms.^2^ Anti-MDA5 (melanoma differentiation-associated gene 5) autoantibody-positive dermatomyositis (DM) constitutes a distinct subtype of myositis. It is characterized by skin ulceration and rapidly progressive interstitial lung disease (RP-ILD), which is associated with a high mortality rate of approximately 50%.^2^ Of note, the prevalence of MDA5+ myositis is significantly higher in East Asia, where it is markedly associated with interstitial lung disease (ILD) and a high frequency of RP-ILD.^2^^,^^3^ The current treatment of MDA5+ myositis with conventional glucocorticoids and immunosuppressants shows limited efficacy, resulting in a poor overall prognosis. This highlights the critical need to investigate the underlying molecular mechanism of MDA5+ myositis and to explore innovative therapeutic strategies.

The precise etiology and pathogenesis of MDA5+ myositis remain incompletely understood. The disease is thought to be initiated by complex interactions between genetic susceptibility and environmental triggers, which break immune tolerance and lead to the production of anti-MDA5 autoantibodies. Multiple factors, including anti-MDA5 autoantibodies, immune cells, and pro-inflammatory mediators, are implicated in the immunopathogenesis of MDA5+ myositis.^4^ A well-known feature of various DM serotypes, including MDA5+ DM, is the upregulation of type I interferon (IFN-I)-inducible genes, which has been observed in the skin, lungs, muscle, and peripheral blood of these patients. Interestingly, there is minimal or absent clinical muscle involvement in the MDA5+ patients.^4^ Previous studies showed only minimal histopathological anomalies of MDA5+ muscles, with scarce inflammation, focal perimysial macrophage infiltration, absent or focal MHC-I expression, and rarely observed complement deposition, compared to other DM serotypes.^5^

Recent advances in single-cell (sc) profiling have enabled in-depth cellular/molecular characterization of myositis. scRNA-seq studies of peripheral blood mononuclear cells (PBMCs) from MDA5+ patients revealed increased proportions of immune cells alongside an overactivated IFN-I response.^6^^,^^7^ However, how immune dysfunction and immune cells mediate inflammation and affect the muscles of MDA5+ patients remains largely unknown. In this study, we performed spatial transcriptomic profiling on muscle biopsies from a cohort of patients with myositis and healthy controls, aiming to delineate the MDA5+ DM-specific molecular, cellular, and microenvironmental signatures.

## Results

### Spatial transcriptomics reveals a distinct gene expression profile in MDA5+ muscles

Spatial transcriptomic sequencing of muscle biopsies collected from 3 MDA5+ DM patients, 8 MDA5- IIM patients (including 2 anti-Ro52, 1 anti-TIF1, 1 anti-Mi2, 1 anti-HMGCR, 1 anti-Ku, and 2 seronegative samples), and 3 healthy controls (Ctrl) were performed (Figures 1A and S1A and Table S1). Phenotypically, the 11 IIM patients included 4 cases of clinically amyopathic DM (CADM) (3 MDA5+), 3 overlap myositis (OM), 2 DM, 1 polymyositis (PM), and 1 immune-mediated necrotizing myopathy (IMNM) (Table S1). Using the Space Ranger pipeline, a total of 9,112 spots were detected from all samples. The data were of high quality, with 15614 median UMI counts and 3774 median genes per spot detected (Figure S1B and Table S2). Spatial Uniform Manifold Approximation and Projection (UMAP) analysis of all samples revealed two distinct groups (Figure 1B). Group 1 included the 3 Ctrl samples, while group 2 included the 3 MDA5+ samples (CADM42, CADM63, CADM76), demonstrating a clear separation of the MDA5+ from Ctrl (Figure 1C). UMAP evaluation confirmed that global separation was driven by biological status rather than technical variance (Figure S1C). Samples co-processed on the same capture area cleanly segregated by group, ruling out slide-specific bias, and any residual batch effects were computationally removed using Harmony.^8^ The OM (OM49, OM60, OM64) and DM (DM70, DM81) samples, however, were distributed across both groups (Figure 1D), suggesting substantial heterogeneity. Pseudobulk analysis was performed to identify differentially expressed genes (DEGs) between MDA5+ vs. Ctrl. Despite the absence of clinical weakness (Table S1), we found immune-related genes such as *ISG15*, *IFI6*, *MX1*, *CMPK2*, *OAS1*, *RSAD2*, etc. were highly enriched in the MDA5+ muscles (Table S2). The Gene Ontology (GO) enrichment analysis of the DEGs upregulated in MDA5+ samples revealed enriched GO terms related to “defense response to virus,” “negative regulation of viral process,” “cellular response to IFN-I,” etc. (Figure 1E and Table S2). The upregulated DEGs in all myositis samples were also enriched for GO terms related to immune responses when compared to the Ctrl (Figure 1F and Table S2). Similarly, when comparing each subtype to the Ctrl, all subtypes were enriched for immune-related pathways (Figures S1D–S1F and Table S2). Specifically, the top 10 frequently upregulated IFN-related genes in DM reported before^9^ (*ISG15*, *IFI6*, *MX1*, *RSAD2*, *MX2*, *OAS1*, *IRF9*, *IFITM1*, *CMPK2*, *OAS3*) were significantly upregulated in the 3 MDA5+ (highlighted in red) and the DM81 samples (Figure 1G). For instance, a robust *ISG15* upregulation was observed (Figure 1H).

**Figure 1:**
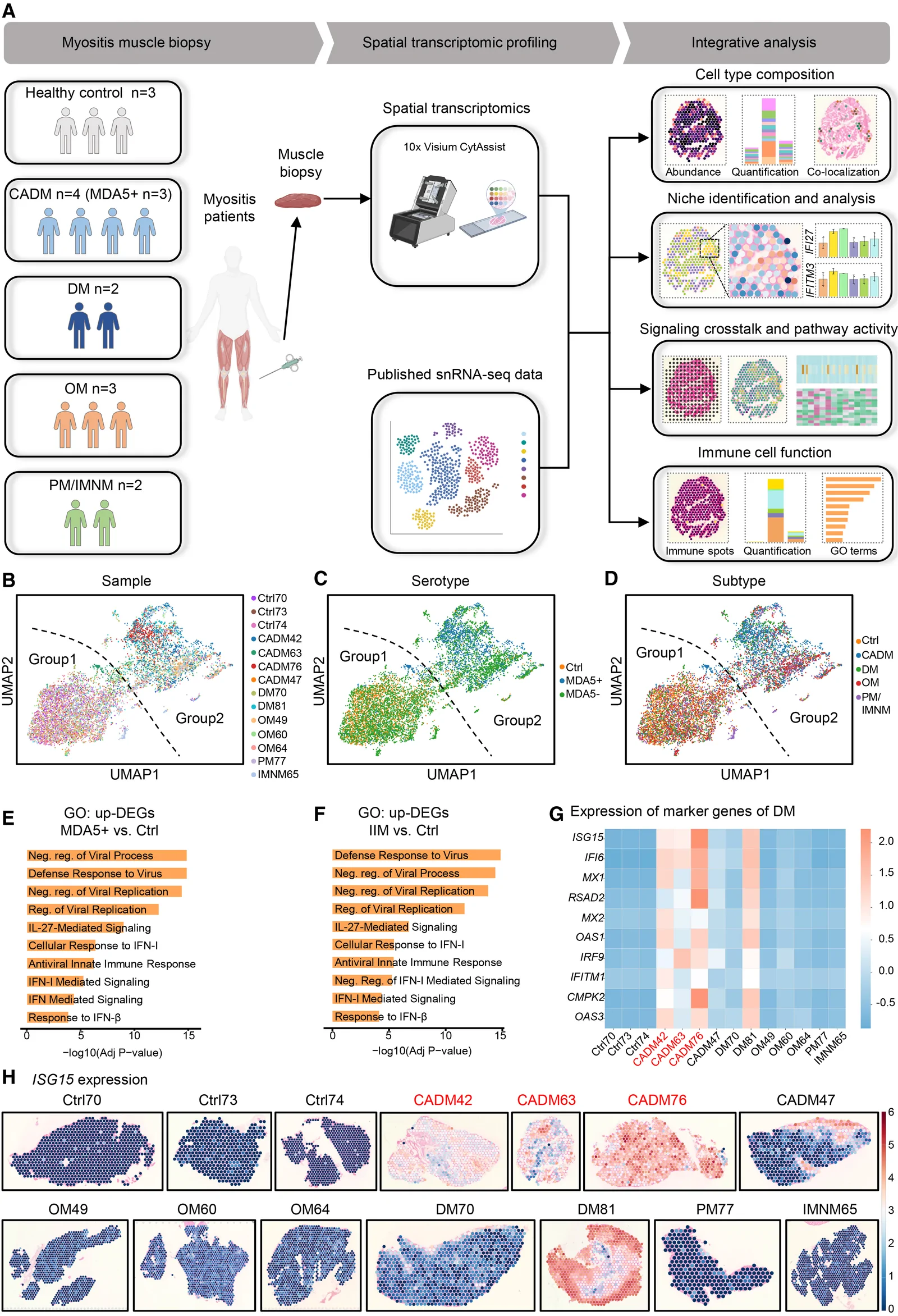
Spatial transcriptomics reveals a distinct gene expression profile in MDA5+ muscles (A) Schematic showing the design of the study. Muscle biopsies were collected from 11 donors with myositis, including 3 MDA5+, 8 MDA5- IIM patients, and 3 healthy controls. Spatial transcriptomics profiling was performed using Visium for fresh-frozen samples and CytAssist (10× Genomics). One publicly available snRNA-seq dataset was adopted for cell type deconvolution analysis. Integrated data analysis was performed, including niche identification, niche-enriched DEG analysis, signaling crosstalk, pathway activity, and immune cell spot analysis. (B) UMAP visualization shows Visium spots of each sample. (C) UMAP visualization shows Visium spots of each serotype. (D) UMAP visualization shows Visium spots of each subtype. (for B–D, the dashed line separating the two major groups of spots was manually added as a visual aid to highlight the global transcriptomic divergence between the two groups.) (E) GO plot of biological process terms enriched in MDA5+ groups (*n* = 3) using the top 200 upregulated genes, compared to the Ctrl group (*n* = 3). The x axis shows -log10 adjusted *p* value. (F) GO plot of biological process (BP) terms enriched in myositis groups (IIM) (*n* = 11) using the top 200 upregulated genes, compared to the Ctrl group. The x axis shows -log10 adjusted *p* value. (G) Heatmap showing the mean expression level of DM marker genes in each sample. The selected marker genes were curated based on previous studies.^9^ The mean expression level was transformed into a row *Z* score for visualization. (H) Spatial gene expression of *ISG15* in all samples; raw counts were log-transformed and normalized.

### Cell type analysis reveals increased immune cell infiltration in MDA5+ muscles

To further elucidate the pathological alterations in MDA5+ muscles, we dissected the cell type composition of the spatial spots by conducting deconvolution analysis using cell2location.^10^ A publicly available single-nuclei RNA-seq dataset derived from IMNM, IBM, and healthy muscles was used for cell type annotation.^11^ The data analysis pipeline and specific cell type annotations were fully reproduced from the original study. A total of 22 cell types were identified (Figures S1G–S1I), including major cell types such as type 1 and type 2 myonuclei (type 1/2 MN), stressed myonuclei (sMN), reactive myonuclei (rMN), endothelial cells (ECs), pericytes, fibro-adipogenic progenitors (FAPs), skeletal muscle stem cells (MuSCs), and immune cells. Estimated cell type abundance was quantified, and a high heterogeneity in cell composition was observed among the myositis samples (Figure 2A and Table S3). For example, the abundance of sMN (from 20% to 56%) and immune cells (from 1.3%–13%) varied across myositis muscles and within a subtype (20% sMN in CADM63 vs. 45% in CADM76, and 32% sMN in OM49 vs. 56% in OM64) (Figure 2A). Quantitative analysis revealed that while MDA5- muscles had a significantly higher proportion of sMNs compared to controls, MDA5+ muscles displayed a milder phenotype (Figure S2A), which was in line with the normal muscle power and CK level in the MDA5+ patients (Table S1). Interestingly, despite the milder myofiber damage, MDA5+ muscles maintained a substantial proportion of immune cells (mean 10% vs. 4% in MDA5-) (Figures 2B and S2A). Across clinical subtypes, OM and DM muscles exhibited a numerically higher trend in sMN abundance (Figures 2C and S2B).

**Figure 2:**
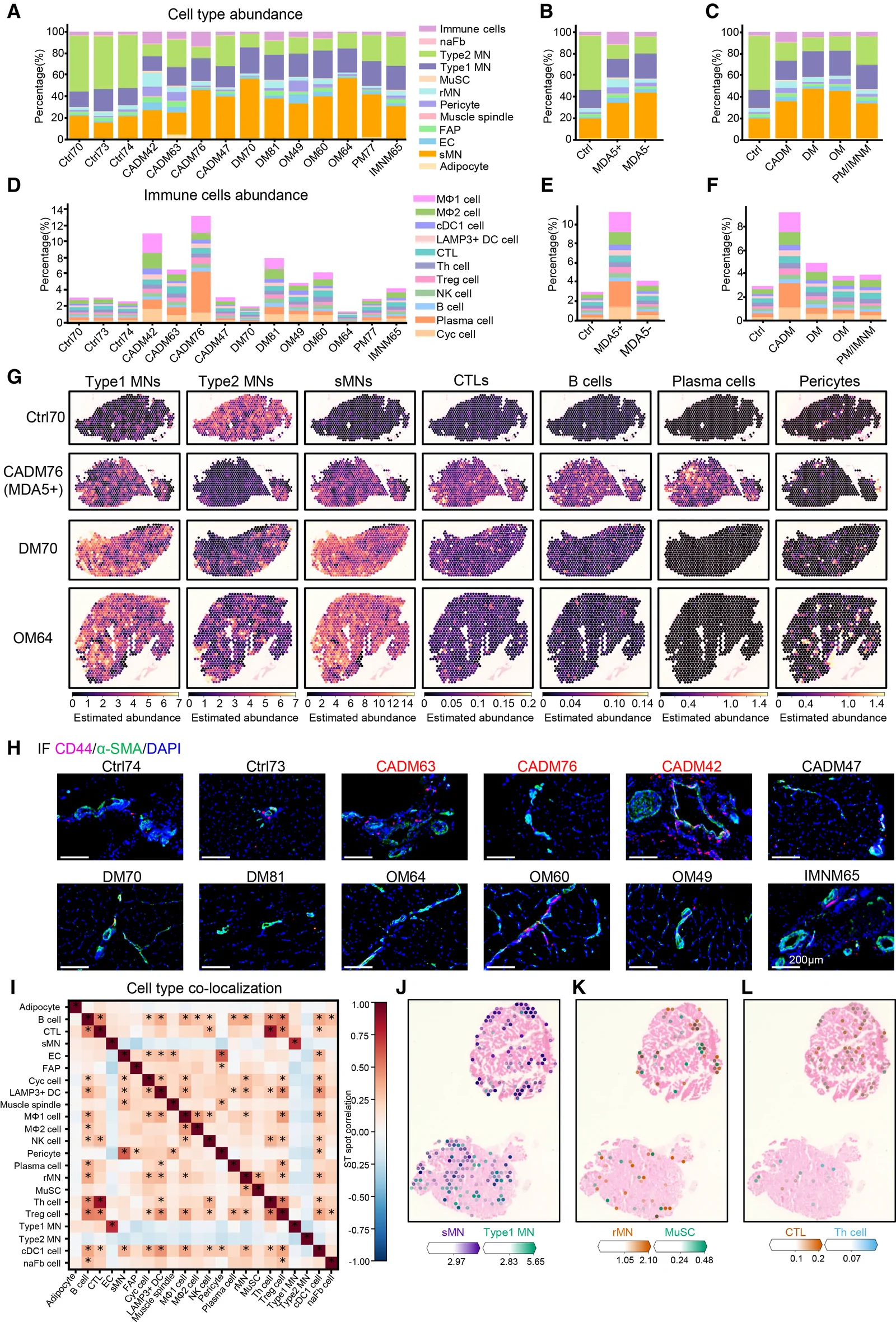
Cell type analysis reveals increased immune cell infiltration in MDA5+ muscles (A) Cell type composition in each sample, calculated by deconvolution of cell types. (B) Comparison of cell type composition in Ctrl, MDA5+, and MDA5- groups. (C) Comparison of cell type composition in myositis subtypes. (D) Abundance of immune cell types in each sample. (E) Comparison of immune cell abundance in Ctrl, MDA5+, and MDA5- groups. (F) Comparison of immune cell abundance in each myositis subtype. (G) Cell type composition of type 1 myofiber nuclei (type 1 MN), type 2 myofiber nuclei (type 2 MN), stressed myofiber nuclei (sMN), cytotoxic T cells (CTLs), B cells, plasma cells, and pericytes in 4 representative samples. (H) Immunofluorescence staining of CD44 (red) in representative samples. Nuclei were stained with DAPI (blue), and blood vessels were stained with α-SMA (green). Scale bars, 200 μm. (I) Heatmaps showing the correlation of spatial locations of cell types (∗Pearson’s correlation coefficient > 0.25). (J–L) Spatial plots show the abundance and co-localization of sMNs and type 1 MN (J), rMN and MuSC (K), CTL and Th cell (L) in Ctrl73 and CADM63 samples.

We then examined the immune cell types closely; a total of 11 types of immune cells were identified, including type 1 and type 2 macrophages (MΦ1, MΦ2), canonical type 1 dendritic cells (cDC1 cells), LAMP3+ dendritic cells (LAMP3+ DC cells), cytotoxic T cells (CTLs), helper T cells (Th cells), regulatory T cells (Treg cells), natural killer cells (NK cells), B cells, plasma cells, and cycling immune cells (Cyc cells) (Figures 2D and S3A). Despite notable inter-sample heterogeneity (Figure S2A), deconvolution analysis revealed an enrichment of specific immune infiltrates—including LAMP3+ DCs, Tregs, and NK cells—in the MDA5+ group relative to MDA5- muscles (Figures 2E, S2A, and S3B). Similarly, the CADM subtype displayed elevated proportions of LAMP3+ DCs compared to DM and OM (Figures 2F, S2B, and S3C). For instance, as shown in Figure 2G, the MDA5+ CADM76 muscle showed an increased abundance of B cells, plasma cells, and CTLs compared to the MDA5- muscles (DM70 and OM64) but reduced abundance of sMN. To validate the abovementioned finding, we stained for CD44 (a marker for immune cells and infiltration) and found increased signals in the myositis muscles, especially in the vascular regions of the two MDA5+ muscles (CADM63 and CADM42) and OM60 muscles (Figures 2H and S3D). Co-staining with specific immune cell markers (CD68, CD20, CD163, and CD11c) alongside the endothelial marker CD31 was also performed (Figures S3E–S3H). We observed a substantial increase in the numbers of these immune cells in the MDA5+ muscles, confirming that most of these cells were located in close proximity (200 μm) to the vasculature.

Furthermore, we dissected the spatial co-localization of cell types across all samples (Figure 2I) and found that sMN and type 1 MN frequently co-occupied the same spatial spots, suggesting that type 1 fibers may be particularly sensitive to stress (Figures 2I and 2J). MuSCs frequently co-localized with rMN, highlighting their role in repairing the muscle fibers (Figures 2I and 2K). The Th cells were found to co-localize with CTLs, possibly facilitating CTL activation (Figures 2I and 2L). B cells exhibited a significant co-localization with CTLs (Figures 2I and S3I) alongside Th and cDC1 cells (Figure 2I). Expectedly, ECs and pericytes co-localized at the blood vessels (Figure S3J). Type 1 and type 2 MN displayed a negative correlation and were distributed separately (Figure S3K).

### Niche analysis reveals a distinct cellular microenvironment in MDA5+ muscles

To further examine the complex microenvironment in the myositis muscles, we then identified cellular niches defined by the cell type composition of spatial spots. Non-negative matrix factorization (NMF) analysis was conducted based on the deconvoluted cell types of each spot (Figure 3A). Consequently, 6 types of niches were identified, the “EC niche” (dominated by ECs and pericytes), “FAP niche” (dominated by FAPs), “rMN niche” (consisted of rMN, MuSCs, and immune cells), “type 1 MN niche” (dominated by type 1 MN), “type 2 MN niche” (dominated by type 2 MN), and “sMN niche” (consisted of sMN and immune cells such as B cells, plasma cells, and Treg cells) (Figures 3B and 3C). For instance, the quantification of cell compositions of sMN niches showed dominance by sMNs (Figures 3D and 3E). Furthermore, DEG analysis revealed distinct expression profiles of each niche type, and the top-ranked DEGs were consistent with their cell type compositions (Figure 3F and Table S4). For example, EC markers *VWF*, *CDH5*, and *PECAM1*, smooth muscle cell markers *MYH11*, *ACTA2*, and *TAGLN* were highly expressed in the EC niche; FAP markers *DCN*, *FBN1*, *MFAP5*, and *PCOLCE* were highly expressed in the FAP niche; muscle regeneration related genes *MYOG* and *MYH3* were highly expressed in the rMN niche; autophagy related gene *SQSTM1*, stress response genes *MKNK2*, *MTLN*, *SELENOM*, *CRYAB*, and *NFE2L1* were upregulated in the sMN niche; fast skeletal muscle fiber-associated genes *MYH1*, *MYH2*, *TNNT3*, and *TNNI2* were enriched in type 2 MN niche, while slow-twitch fiber-associated genes *MYH7B*, *MYH7*, and *ATP2A2* were enriched in the type 1 MN niche (Figure 3F). Expectedly, a limited number of sMN niches were detected in the 3 Ctrl muscles, but a much higher number were found in the myositis muscles (Figures 3G and 3H). A lower percentage of sMN niches was also detected in the MDA5+ vs. MDA5- muscles (33% vs. 41%) (Figure 3I). When comparing subtypes, a higher proportion of the sMN niche was observed in DM (58%) and OM (50%), compared to 31% in CADM (Figure 3J). The niche heterogeneity was also detected within subtypes. For instance, 25% of the sMN niches were found in CADM47 vs. 4% in CADM63, and 31% in DM81 vs. 86% in DM70 (Figures 3G and 3H).

**Figure 3:**
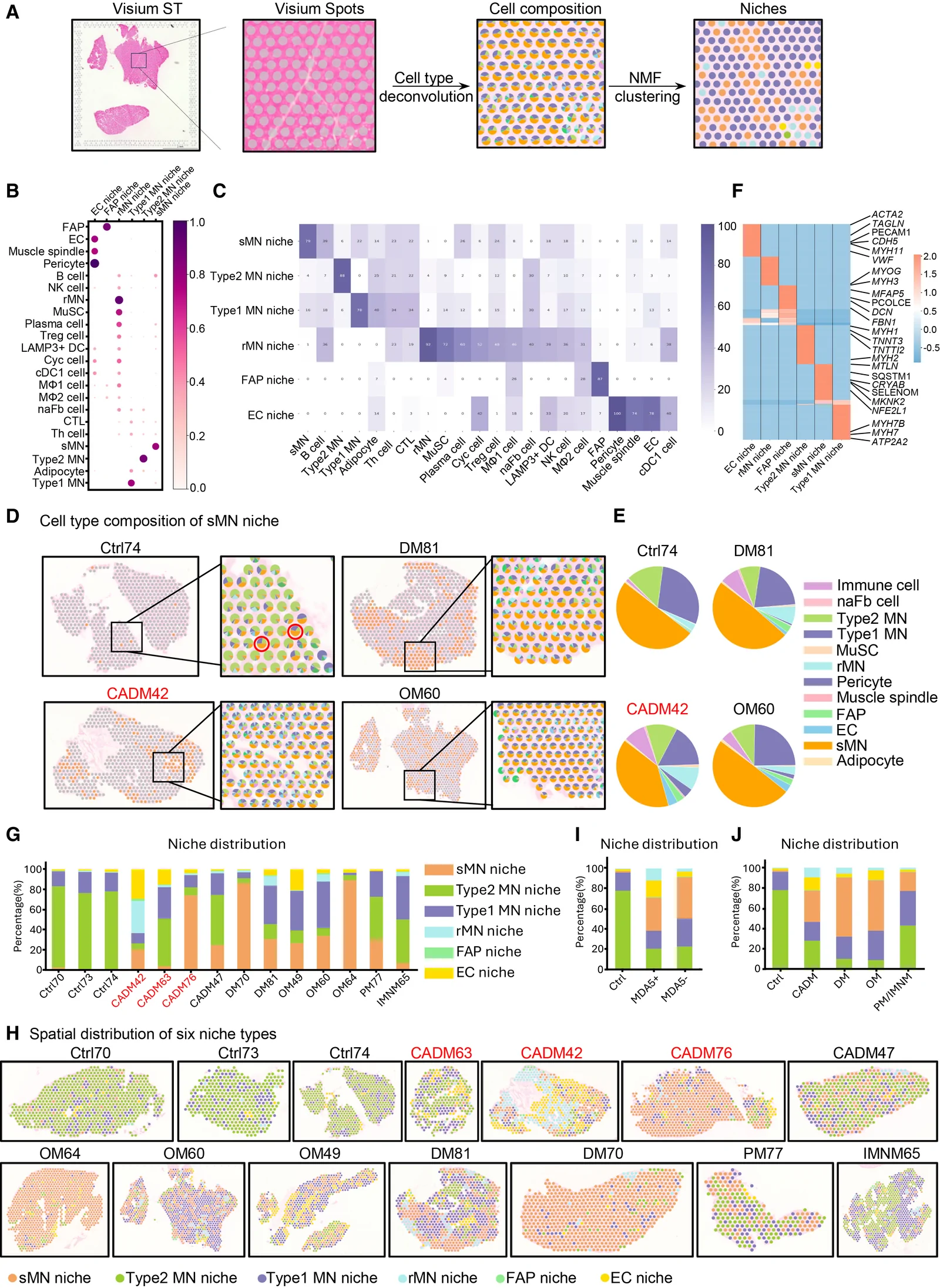
Niche analysis reveals a distinct cellular microenvironment in MDA5+ muscles (A) Schematic illustration of the niche identification with non-negative matrix factorization (NMF) analysis based on cell type composition. (B) Dot plot of the estimated NMF weights of cell types (rows) across niche types (columns). (C) Heatmap shows the normalized distribution of estimated cell type abundance in percentages across the niches. (D) Illustration of the “sMN niche” dominated by sMNs in four representative samples. (E) Pie chart shows the cell type composition of the “sMN niche.” (F) Heatmap shows the top-ranked 50 DEGs in each niche type. (|log2FC| > 1.3, adjusted *p* < 0.05). (G) Bar plot shows the niche composition in all samples. (H) Spatial plot shows the distribution of six types of niches across all samples. (I) Bar plot comparison of the niche composition in Ctrl, MDA5+, and MDA5 groups. (J) Bar plot comparison of the niche composition in each subtype.

### Unique gene expression in the EC niche of MDA5+ muscles

To evaluate the molecular changes of the MDA5+ muscles, we analyzed DEGs in the above-defined niches (Table S5). When comparing type 1 and type 2 MN niches in the MDA5+ vs. MDA5- muscles, the up-DEGs were both enriched for GO terms related to “cellular response to IFN-I,” “IFN-I mediated signaling pathway,” “defense response to virus,” etc., and the down-DEGs were enriched for “neuromuscular junction development,” “actomyosin organization,” etc. (Figures 4A and 4B). When comparing MDA5+ vs. MDA5- sMN niches, the up-DEGs were also enriched for similar IFN-related GO terms (Figure 4A). For example, IFN-stimulated gene, *OASL*, was upregulated in type 1 MN, type 2 MN, and sMN niches in the MDA5+ muscles (Figure 4C). *NUPR1*, a stress-response gene that is a critical repressor of ferroptosis,^12^ was upregulated in all myositis samples but showed a higher expression in the MDA5+ muscles (Figure S4A). When comparing the rMN niches of MDA5+ vs. MDA5- muscles, the up-DEGs were enriched for “extracellular matrix organization,” “collagen fibril organization,” etc. (Figure S4B). The up-DEGs in the FAP niches were enriched for “ECM pathways,” while “sarcomere organization” and “myofibril assembly” related pathways were downregulated (Figure S4C).

**Figure 4:**
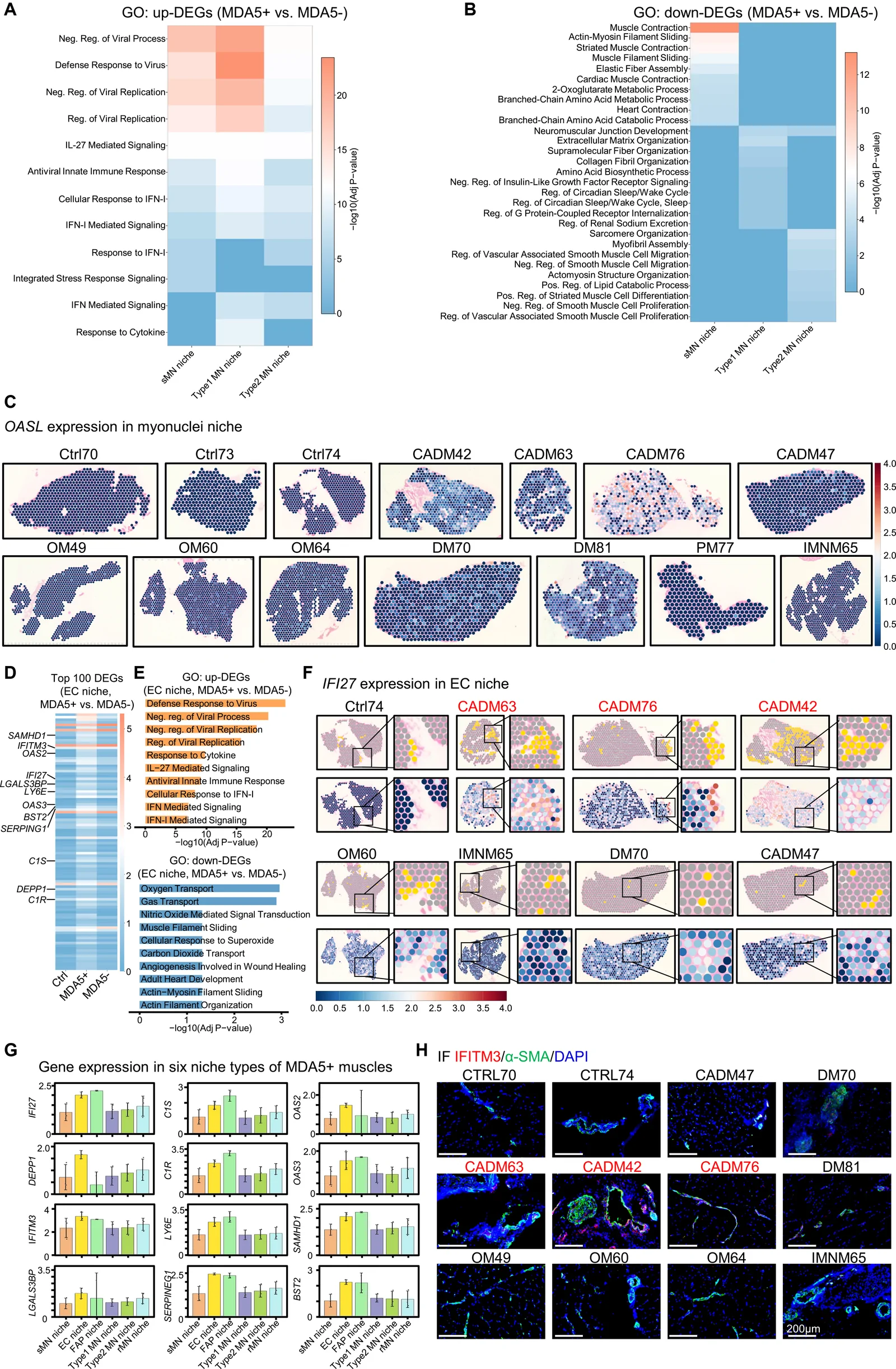
Unique gene expression in the EC niche of MDA5+ muscles (A) Heatmap showing the top 10 enriched GO terms of upregulated genes in the sMN niche, type1/2 MN niche, colored by –log10 adjusted *p* value. (B) Heatmap shows the top 10 enriched GO terms of down-regulated genes in the sMN niche, type1/2 MN niche, colored by –log10 adjusted *p* value. (C) Normalized spatial expression of *OASL* in all samples. (D) Heatmap shows the top 100 DEGs in the EC niche of Ctrl, MDA5+, and MDA5- groups. (|log2FC| > 1.3, adjusted *p* < 0.05). (E) Top 10 enriched GO terms of up- and down-regulated genes in the EC niche of MDA5+ vs. MDA5- muscles. The x axis shows -log10 adjusted *p* value. (F) Spatial plots show the distribution of EC niches (yellow spots) and the expression of *IFI27* in representative Ctrl, MDA5+, and MDA5- muscles. (G) Bar plots show the expression of genes enriched in the EC niche of MDA5+ muscles. Data are represented as the mean ± SD. Dots represent the mean expression of each patient. (H) Immunofluorescence staining of IFITM3 (red) and α-SMA (green) in representative myositis muscles. Nuclei were stained with DAPI (blue). Scale bars, 200 μm.

Notably, when comparing the MDA5+ vs. MDA5- EC niches, the up-DEGs were enriched for anti-viral responses and responses to IFN-I-related pathways, while the “oxygen transport,” “gas transport,” “nitric oxide mediated signal transduction,” and “carbon dioxide transport” pathways were enriched in the down-DEGs, indicating increased immune response and dampened oxygen/gas exchange function of blood vessels of the MDA5+ muscles (Figures 4D and 4E). For example, *IFI27* gene expression was upregulated in MDA5+ vs. MDA5- EC niches (Figures 4D and 4F). Of note, the expression of *IFI27* also showed an enrichment in the EC niches compared to other myonuclei niche types in the MDA5+ muscles (Figures 4F and 4G). Similarly, a group of up-DEGs in the MDA5+ EC niche, including IFN-I-stimulated genes (*IFITM3*, *OAS2*, *OAS3*, *LY6E*, and *BST2*), anti-viral immune-related genes (*LGALS3BP* and *SAMHD1*), complement system-related genes (*SERPING1*, C*1S*, and *C1R*), and autophagy-related gene *DEPP1*, were also enriched in the EC niche of MDA5+ muscles, compared to other myonuclei niche types of MDA5+ muscles (Figure 4G). This was further validated by immunofluorescence staining of IFITM3, SAMHD1, IFI27, and BST2, co-staining with α-SMA to label the blood vessels; increased staining was found in the blood vessel regions in the MDA5+ muscles (Figures 4H and S4D–S4F). We then examined the source of IFNs that activate the expression of these IFN-I inducible genes in MDA5+ muscles. Surprisingly, type I, II, and III IFN transcripts were barely detectable in the Ctrl or myositis muscles, but several IFN receptors, such as *IFNAR1/2* and *IFNGR1/2*, were upregulated (Figure S4G). Together, the analyses discovered a cohort of proinflammatory and IFN-I inducible genes enriched in the EC niche of MDA5+ muscles, highlighting the prominent IFN-I signature and vasculopathy of MDA5+ muscles.

The transcriptomic differences of each niche type were also analyzed among OM and DM subtypes. The OM muscles showed a reduced response to IFN-I in sMN, EC, type 1, and rMN niches, compared to other subtypes, while the DM muscles exhibited the known IFN-I signature in EC, type 1, and type 2 niches (Figure S5).

### Cellular crosstalk analysis uncovers enriched signaling pathway activities in the EC niche of MDA5+ muscles

Next, to dissect the cell-cell communication between niches, we analyzed the crosstalk signals using the COMMOT method.^13^ A total of 42 signaling pathways were detected, and most of them were heightened in myositis muscles (Figures 5A and S6A and Table S6). For instance, several signaling have been reported to be upregulated in myositis, including CCL,^14^ CSF,^14^ galectin-9-CD44,^15^^,^^16^ MIF,^17^ COMPLEMENT,^18^ CXCL,^14^^,^^16^ granulin (GRN),^19^ PDGF,^20^ VEGF,^20^^,^^21^ and Visfatin.^22^ Among these, our analysis revealed that TGF-β signaling was upregulated in myositis muscles compared to healthy controls (Figures 5B and 5C). Furthermore, we found that sMN niches secreted the highest amount of TGF-β signaling molecules (Figure 5D), consistent with the report that TGF-β could be secreted from “inflammatory myonuclei”.^23^ We also analyzed the published scRNA-seq data^11^ using CellChat, which consistently revealed extensive cellular crosstalk mediated by TGF-β, MIF, COMPLEMENT signaling, etc. (Figure S7).

**Figure 5:**
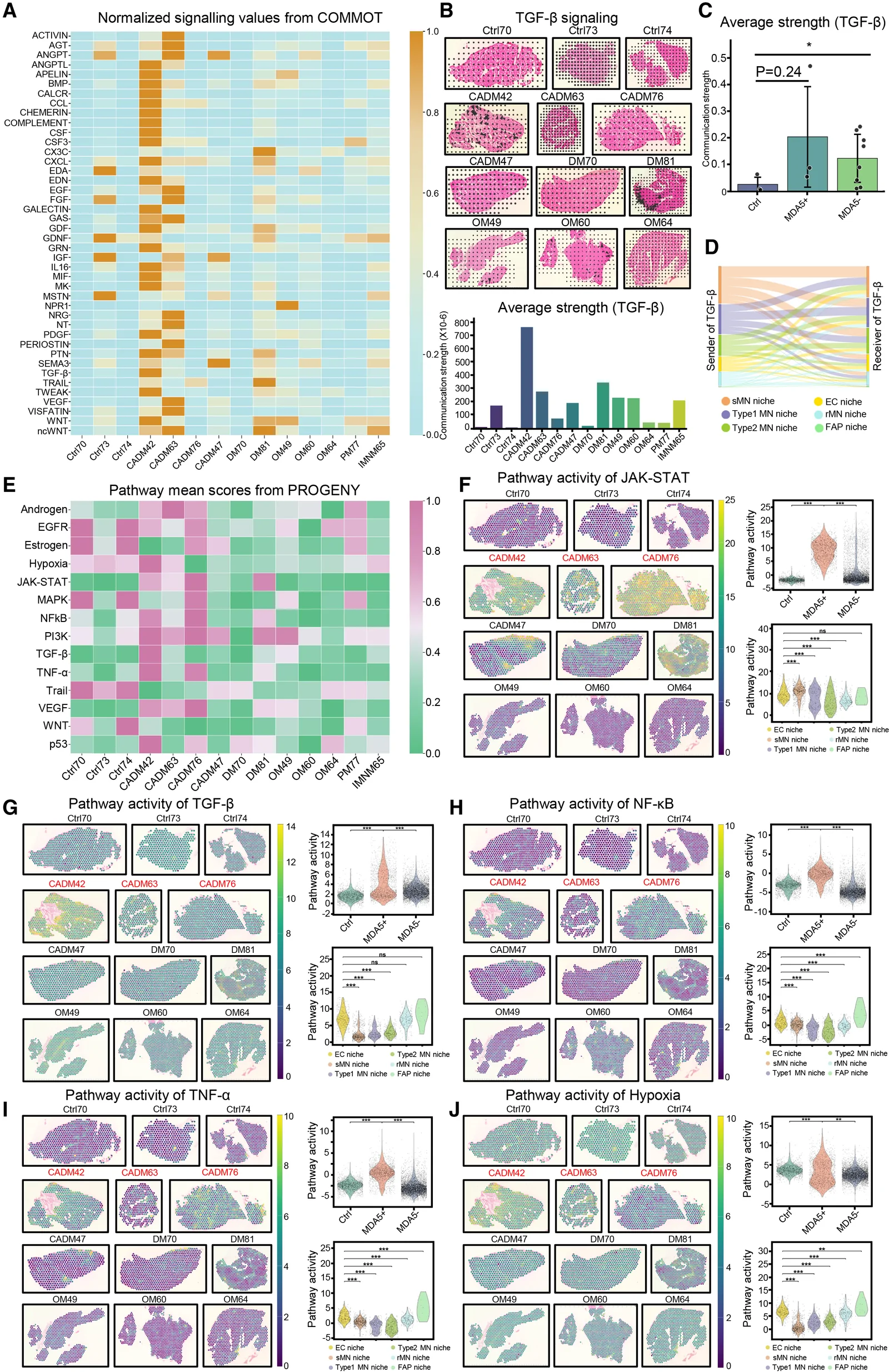
Cellular crosstalk analysis uncovers enriched signaling pathway activities in the EC niche of MDA5+ muscles (A) Heatmap shows the normalized signaling communication strength calculated by COMMOT in all samples. (B) The signaling direction (top) and the normalized signaling communication strength (bottom) in each sample are shown for TGF-β signaling. (C) Comparison of the normalized signaling communication strength for TGF-β signaling in the Ctrl, MDA5+, and MDA5- groups. Dots represent the mean strength of individual MDA5+ patient. Data are represented as the mean ± SD. Statistical significance was determined using Student’s *t* test. (D) Sankey plot shows the secreting and receiving niche of the TGF-β signaling. (E) Heatmap shows the normalized pathway activity scores calculated by PROGENY in all samples. (F) Normalized activity scores of the JAK-STAT pathway in representative samples (left). Violin plot shows the comparison of JAK-STAT activity scores in the Ctrl (*n* = 3), MDA5+ (*n* = 3), and MDA5- groups (*n* = 8) (right-top). Violin plot shows the JAK-STAT activity scores in each niche type of MDA5+ muscles (right-bottom). (G–J) The previous analyses were performed for the TGF-β (G), NF-κB (H), TNF-α (I), and hypoxia (J) pathways (∗*p* <0.05; ∗∗*p* <0.01; ∗∗∗*p* <0.001). Statistical significance was determined using the Mann-Whitney *U* test.

To strengthen the abovementioned results, we examined the downstream gene expression of signaling pathways by PROGENY analysis (Figure 5E and Table S6). As a result, JAK-STAT pathway activities were upregulated in the 3 MDA5+ and the CADM47, DM81 muscles (Figures 5E and S6B), and the MDA5+ muscles showed significantly higher activity compared to MDA5-muscles (Figures 5F and S6C). Moreover, the activity of the TGF-β pathway was upregulated in the MDA5+ muscles, together with increased NF-κB, TNF-α, hypoxia, PI3K, VEGF, and p53 pathways compared to MDA5- muscles (Figures 5G–5J and S6D). Furthermore, we examined the pathway activities in each niche type. Notably, the activities of TGF-β, hypoxia, NF-κB, and TNF-α pathways were enriched in the EC niches of MDA5+ muscles compared to the other myonuclei niche types (Figures 5G–5J), which is consistent with the result showing increased immune responses and dampened oxygen/gas transport function in the EC niches (Figure 4E). To address inter-sample variability, we also performed a sample-level pseudo-bulk analysis. In this global analysis, broadly upregulated pathways including JAK-STAT, PI3K, and VEGF retained statistical significance in the MDA5+ group (Figures S6C and S6E), compared to Ctrl and MDA5- groups. Furthermore, consistent with our spot-level observations, the TGF-β, NF-κB, TNF-α, and hypoxia pathways maintained an upward trend. While these latter pathways did not reach statistical significance at the sample level—likely due to the dilution of highly localized perivascular signals by the surrounding unaffected tissue in the bulk average—the enrichment of these pathways within the EC niche was maintained in the MDA5+ muscles when assessed at the individual sample level (Figure S6C).

### Macrophages associated with humoral response in MDA5+ muscles

The infiltration of immune cells in myositis muscles has been well documented,^24^^,^^25^^,^^26^ but it is still unclear how the cells promote inflammation and muscle atrophy/damage. To elucidate the functional role of immune cells within specific muscle niches, we first ranked the cell type abundance of all the Visium spots and defined the “immune cell spots” according to the highest immune cell type abundance (the top 5%). For example, spots with the highest 5% of MΦ1 abundance were classified as “MΦ1 spots” (Figure 6A). We also quantified the proportion of these spots in each sample and characterized their associated niche types (Figure 6B and Table S7). Despite substantial inter-sample heterogeneity, MDA5+ and CADM muscles exhibited a generally higher proportion of these high-abundance immune spots compared to other myositis subtypes (Figures 6C, 6D, S8A, and S8B).

**Figure 6:**
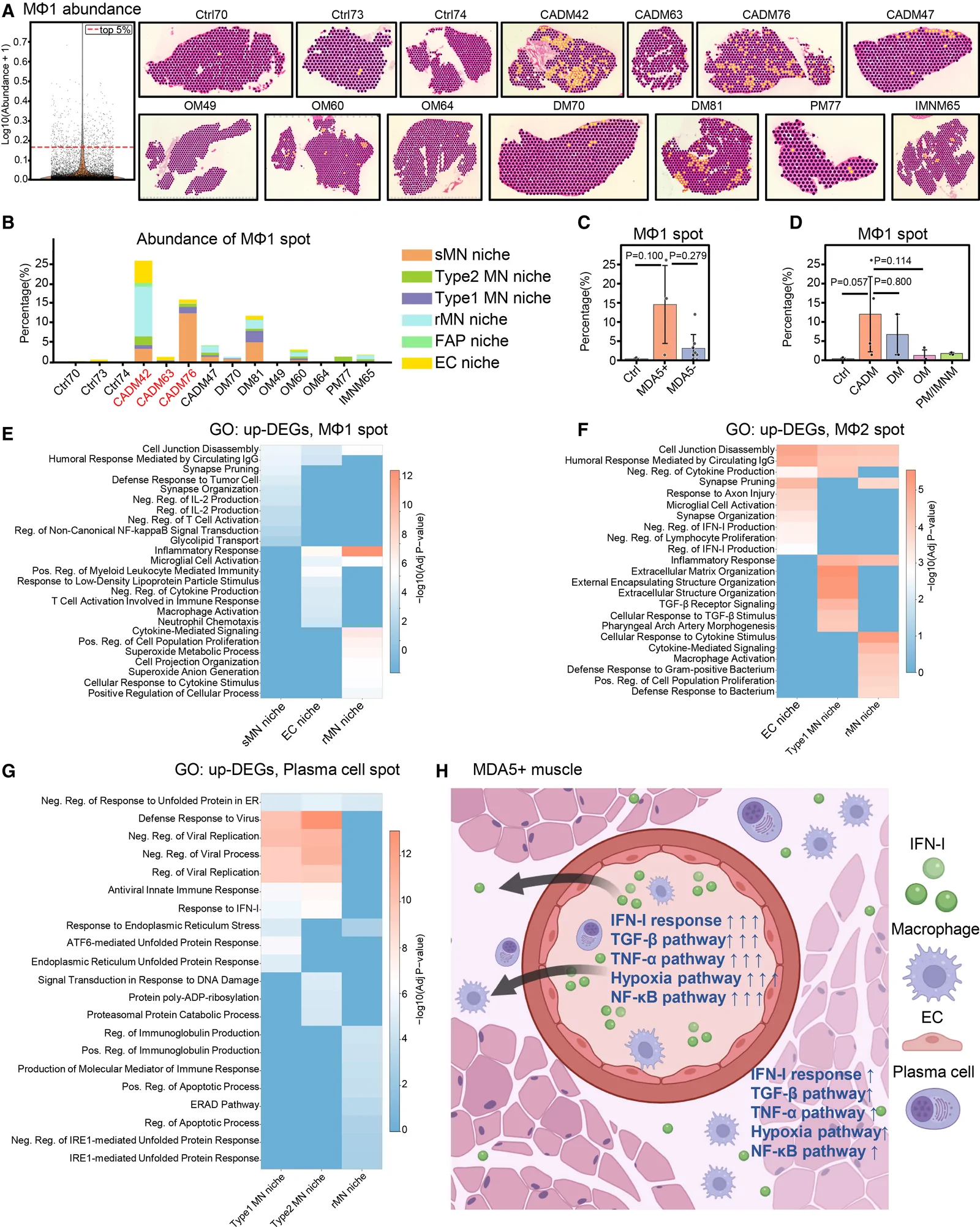
Macrophages associated with humoral response in MDA5+ muscles (A) Left: distribution of MΦ1 abundance across all spots from all samples; the top 5% were defined as “MΦ1 spot.” Right: distribution of MΦ1 spots in each sample. (B) Proportion of MΦ1 spots within each sample, stratified by niche type. (C) Proportions of MΦ1 spots in Ctrl, MDA5+, and MDA5- groups. Data are presented as the mean ± SD, with each dot representing an individual patient. Statistical significance was determined using the Mann-Whitney *U* test. (D) Proportions of MΦ1 spots in each myositis subtype. Data are presented as the mean ± SD, with each dot representing an individual patient. Statistical significance was determined using the Mann-Whitney *U* test. (E) Heatmap shows the GO terms of upregulated genes of MΦ1 spot in the EC, rMN, and sMN niches, colored by–log10 adjusted *p* value. (F) Heatmap shows the GO terms of upregulated genes of MΦ2 spot in the EC, rMN, and type 1 MN niches, colored by –log10 adjusted *p* value. (G) Heatmap shows the GO terms of upregulated genes of Plasma cell spot in the rMN, type 1, and type 2 MN niches, colored by –log10 adjusted *p* value. (H) Schematic summarizes the study. Response to IFN-I is upregulated in the EC niches of MDA5+ muscles, possibly activated by the systemic IFN-I from blood. Pathway activities of TNF-α, NF-κB, hypoxia, and TGF-β are also enriched in the EC niches. Immune cells, such as macrophages and plasma cells, infiltrate into muscles via blood vessels.

To elucidate the functional impact of immune cells, we compared gene expression of the immune cell spots vs. non-immune cell spots in each niche type of MDA5+ muscles. For example, we compared MΦ1 spots vs. non-MΦ1 spots in the sMN niche of MDA5+ muscles and found upregulated pathways, including “humoral immune response mediated by circulating IgG” and “regulation of non-canonical NF-κB signal transduction,” enriched in the MΦ1 spots. Similarly, MΦ1 spots in the EC niches and rMN niches showed upregulated pathways of “humoral immune response mediated by circulating IgG,” “cytokine-mediated signaling,” “T cell activation involved in immune response,” and “inflammatory response,” suggesting that MΦ1 correlated with the humoral response to autoantibodies and immune cell activation in MDA5+ muscles (Figure 6E and Table S7). The MΦ2 spots in the EC niche, type 1 MN niche, and rMN niche also showed upregulated pathways of “humoral immune response mediated by circulating IgG” (Figure 6F). Furthermore, antiviral response, immunoglobulin production, and endoplasmic reticulum stress-related pathways were found to be upregulated in the plasma cell spots (Figure 6G). Moreover, we found Treg cell spots in the type 1 MN niche associated with negative regulation of immune response, and LAMP3+ DC cell spots in type 2 MN niche were associated with upregulated IFN-I and antiviral response (Figures S8C and S8D). Collectively, the abovementioned findings illuminate the functional impact of spatially localized immune cells, highlighting a unique immunopathological association in MDA5+ muscles wherein macrophages associate with humoral immune response.

## Discussion

MDA5+ myositis is a distinct subtype of myositis characterized by scarce or absent muscle involvement and prominent involvement of skin and lungs. To enhance our understanding of the molecular underpinnings of the disease, we profiled the spatial transcriptome of muscle biopsies collected from a cohort of IIM patients composed of various phenotypes and serotypes, including 3 rare samples from MDA5+ CADM patients. The UMAP reduction separated the MDA5+ muscles well from the healthy muscles, suggesting a distinct spatial transcriptomic profile of the MDA5+ muscles. Next, we analyzed the cell type composition and found an unexpected increase in immune cells, as well as a decrease in stressed MN in the MDA5+ muscles. This increased immune cell infiltration could be explained by immune responses and damage to blood vessels, leading to local inflammation and enhanced infiltration of immune cells through the wall of the vessel into the perivascular regions. In line with this, we found that staining for CD44, a marker for immune cells that also functions in cell migration as an adhesion molecule, was also enriched at the vascular regions, alongside an increase in immune cell subsets. Unlike IBM or IMNM, in which the CD8^+^ cytotoxic T cells directly target muscle fibers, the increased immune cells are focal in the vascular region of MDA5+ muscles, which may not directly target muscle fibers, explaining why fewer stressed MN were detected in MDA5+ muscles. Furthermore, we found the stressed MN colocalized with aerobic type 1 fibers that are more sensitive to hypoxia, possibly caused by EC damage, compared to anaerobic type 2 fibers.

Spatial transcriptomics overcomes the limitations of bulk sequencing by resolving gene expression within specific tissue regions/niches. In MDA5+ muscles, our data provide a transcriptome-wide view of six characteristic niches, with a particular focus on the EC niche (perivascular region). Within this region, we discovered a cohort of IFN-I-induced genes that were upregulated compared to the myofiber region (Figure 4), providing spatially resolved evidence that the well-known IFN signature is not uniformly distributed, but is profoundly enriched within the perivascular microenvironment. Concurrently, we identified a downregulation of oxygen transport-related genes, pointing to vascular dysfunction. More interestingly, we found barely detectable IFN mRNA expression in myositis muscles, suggesting that the IFN may originate from the blood and that the blood vessels respond first to the circulating IFN, before muscle fibers react to the IFN diffused from the blood vessels. This model of a circulation-driven mechanism is highly concordant with recent *in vitro* findings by Kaewin et al.,^27^ who demonstrated that systemic factors within DM patient serum can activate the IFN signaling pathway in skeletal muscles. Consistent with this hypothesis, we found that the expression of the identified group of IFN-I-stimulated genes was upregulated in the EC niches to a greater degree than in the myonuclei niches. Interestingly, ECs in the skin biopsies of DM patients showed strong IFN-I-inducible MX1 and ISG15 staining,^28^^,^^29^ suggesting this EC response to IFN-I is a systemic phenomenon.

Moreover, we analyzed the cell-cell communication and pathway activity and found that hypoxia, NF-κB, TNF-α, and TGF-β pathway activities showed enrichment in the EC niche of the MDA5+ muscles. Consistent with our findings, positive staining of the phosphorylated p65 subunit of NF-κB was found in the blood vessel endothelium of DM patients.^30^ Immunoreactive TNF-α granules were also detected on ECs at perifascicular sites in DM muscles.^31^ TGF-β has been reported to be upregulated in the serum of DM/PM patients complicated with ILD,^32^ as well as in the muscle biopsies of DM and IBM patients.^33^^,^^34^ We speculate that this hypoxic, TGF-β-rich microenvironment drives CD103 expression in tissue-resident memory T (Trm) cells identified in a recent single-cell study of muscle-infiltrating T cells,^35^ reprogramming them into a chronic pathogenic state. Interestingly, a mouse study constitutively activating TGF-β in the ECs resulted in severe cutaneous, pulmonary, and microvascular fibrosis, and loss of ECs due to EndoMT, mimicking the manifestations of MDA5+ DM,^36^ suggesting the role of the TGF-β pathway in promoting MDA5+ disease.

Finally, we analyzed the important role of immune cells in MDA5+ myositis pathogenesis. In the MDA5+ muscles, macrophage spots were associated with upregulation of humoral response to circulating IgG, immune cell activation/proliferation, suggesting that macrophages may play an important role in responding to autoantibodies and promoting inflammation in the disease. However, as our spatial distance analysis (Figure 2I) revealed that macrophages are not in close proximity to plasma cells, whether this response to IgG is driven by locally secreted antibodies or the deposition of systemic circulating IgG requires further investigation. To further validate the specific functional roles of diverse immune infiltrates, *in vitro* co-culture systems utilizing myoblasts/ECs will be highly valuable. These models—similar to those previously established to study dendritic cells and cytotoxic CD28null T cells—allow for the direct evaluation of cell-mediated injury and the interplay between immune cells and the muscle or ECs.^37^^,^^38^ Furthermore, these systems are crucial for assessing targeted treatment effects in MDA5+ DM, such as JAK inhibitors, which have been shown to reduce EC death.^39^

Our results also hint at several potential therapeutic targets for MDA5+ DM. These include the upregulated IFN-I–induced gene program, NF-κB, TNF-α, and TGF-β pathways in the pericyte-EC niche, as well as infiltrating macrophages and plasma cells. They provide mechanistic support for promising new therapies targeting related pathways: JAK inhibition (tofacitinib) in refractory MDA5+ DM,^4^ anti-TGF-β (pirfenidone) in MDA5-associated ILD,^40^ B-cell depletion (rituximab or CD19 CAR-T) in MDA5+ RP-ILD,^41^^,^^42^ and intravenous immunoglobulin (IVIG) in MDA5+ ILD.^43^ While TNF-α inhibition in other myositis subtypes has yielded mixed outcomes,^44^ and NF-κB blockade is being explored in other autoimmune and muscular diseases, our spatial data suggest these pathways are specifically hyperactive in the MDA5+ perivascular niche, providing a rationale for their targeted evaluation. Properly designed clinical trials should be carried out to evaluate these strategies specifically in MDA5+ disease.

In summary, our findings revealed that despite the absence of clinical weakness, MDA5+ muscles exhibited a distinct spatial architecture characterized by enriched IFN-I signature and immune activation in the perivascular region (Figure 6H). While exhibiting a lower burden of stressed myonuclei, MDA5+ muscles showed evident immune infiltration in the perivascular region. Six distinct niche types were identified within myositis muscles, and the EC niche in MDA5+ muscles uniquely demonstrated an upregulation of inflammatory and IFN-I response alongside a downregulation of oxygen transport pathways. Cell-cell communication analysis further suggested heightened hypoxia, TGF-β, TNF-α, and NF-κB signaling, which were particularly enriched within the MDA5+ EC niche. Moreover, functional analysis of immune cells implicated macrophages in driving localized humoral immune responses. Collectively, these findings delineated a unique spatial immunopathology in MDA5+ muscles and revealed potential therapeutic targets.

### Limitations of the study

We must point out that our study was limited to a cohort of only 11 patients due to the rarity of IIM, which restricted our ability to strictly match patient sex across groups. Furthermore, the specific muscle biopsy locations were not perfectly matched due to distinct clinical procurement routes; healthy control samples were collected during orthopedic surgeries, whereas patient samples were obtained via standard conchotome biopsies. Recognizing that baseline structural cell compositions (e.g., fiber types and adipocytes) naturally vary by anatomical site, we focused our abundance analysis on pathological immune cell infiltration rather than resident cell proportions. The bulk composition disparity was mitigated by utilizing the spatial resolution of our data to perform niche-to-niche comparisons. In addition, the small sample size may not be sufficient to investigate the pathogenesis of IMNM or PM phenotypes, or to dissect serotypes other than MDA5+. Furthermore, because all MDA5+ patients in our cohort presented with the CADM phenotype, it is difficult to strictly distinguish features specific to anti-MDA5 from those associated with the CADM phenotype itself. Future studies with larger cohorts are needed to decouple these variables and investigate various subgroups of myositis. Additionally, the snRNA-seq reference dataset used for spatial deconvolution in this study was primarily derived from IMNM, IBM, and healthy muscles. While this is not a perfect pathological match for all subtypes in our cohort, the fundamental cell lineages are shared across inflammatory myopathies, making it possible to map basic cellular compositions.

Building on our findings, several critical avenues for future research emerge. First, the application of single-cell resolution spatial transcriptomics would precisely delineate the cellular changes and signaling pathways within the key vascular and fiber niches we have identified. Second, our inability to detect IFN mRNA within muscle tissues, despite a robust IFN-I signature, suggests an extra-muscular source. It is therefore imperative to determine whether circulating leukocytes (PBMCs) or other affected tissues, such as skin and lung, are the primary contributors of systemic IFN in MDA5+ patients. Furthermore, to obtain a comprehensive understanding of this systemic autoimmune disease, spatial transcriptomic profiling of lung and skin lesions from MDA5+ patients is essential. Such studies would directly reveal the shared and tissue-specific pathways, particularly the systemic alterations in vascular pathology. These mechanistic insights will directly inform therapeutic development.

## Resource availability

### Lead contact

Requests for further information and resources should be directed to and will be fulfilled by the lead contact, Dr. Huating Wang (huating.wang@cuhk.edu.hk).

### Materials availability

This study did not generate new unique reagents.

### Data and code availability

The data that support this study are available from the corresponding authors upon reasonable request. The raw sequence and processed spatial transcriptomics data reported in this paper have been deposited on the Gene Expression Omnibus (GEO) database (GEO: GSE317538) and are publicly available upon publication of this study. Custom scripts described in the Methods will be made available upon request.

## Acknowledgments

This work was supported by the 10.13039/501100012166National Key R&D Program of China to H.W. (project code: 2022YFA0806003); Non-Communicable Chronic Disease-10.13039/501100018537National Science and Technology Major Project of China to H.W. (project code: 2024ZD0530400); The InnoHK initiative of the 10.13039/501100003452Innovation and Technology Commission of the Hong Kong Special Administrative Region Government to H.W.; 10.13039/501100005847Health and Medical Research Fund (10.13039/501100005847HMRF) from 10.13039/100022720Health Bureau of HK to H.W. (project codes: 10210906 and 23242241); 10.13039/100001641General Research Fund (GRF) from Research Grants Council (RGC) of the Hong Kong Special Administrative Region, China, to H.W. (project codes: 14108225, 14103526, 14105123, 14103522, and 14105823); the 10.13039/501100001809National Natural Science Foundation of China (10.13039/501100001809NSFC) to H.W. (project code: 82172436); (1+1+1 CUHK-CUHK(SZ)-GDST Joint Collaboration Fund to H.W. (project code: 524002645); Strategic Topics Grant (STG) from RGC to H.W. (project code: STG1/E-403/24-N); Theme-based Research Scheme (TRS) from RGC to H.W. (project code: T13-602/21-N); Area of Excellence Scheme (AoE) from RGC to H.W. (project code: AoE/M-402/20). Schematic representations (Figures 1A and 6H, and graphical abstract) were created with BioRender.com.

## Author contributions

Y.Q. performed all the experiments; G.L. analyzed all the high-throughput sequencing data; M.O. assisted in the procurement of the healthy human muscle specimens; H. Sun supervised computational analyses; H. So secured the myositis muscle biopsies and associated clinical data. H.W. supervised experiments and data analyses; H.W., H. So, and Y.Q. conceived the project. Y.Q., G.L., H. So, and H.W. wrote the manuscript, with input from all authors.

## Declaration of interests

The authors have declared that no conflict of interest exists.

## Declaration of generative AI and AI-assisted technologies in the manuscript preparation process

During the preparation of the manuscript, Yulong Qiao used “DeepSeek” and “Gemini” to improve language and readability. After using these tools, the authors reviewed and edited the content as needed and take full responsibility for the content of the published article.

## STAR★Methods

### Key resources table

**Table:** 

REAGENT or RESOURCE	SOURCE	IDENTIFIER
**Antibodies**		

Donkey polyclonal anti-Mouse IgG (H + L)	Thermo Fisher Scientific	Cat# A-21202; RRID: AB_141607
Donkey polyclonal anti-Rabbit IgG (H + L)	Thermo Fisher Scientific	Cat# A-21207; RRID: AB_141637
Rabbit multiclonal anti-CD44	abcam	Cat# ab316123; RRID: AB_3665134
Rabbit monoclonal anti-IFITM3	Abclonal	Cat# A24337; RRID: AB_3755777
Rabbit monoclonal anti-SAMHD1	Abclonal	Cat# A4607; RRID: AB_3756321
Rabbit monoclonal anti-BST2	Abclonal	Cat# A23445
Rabbit polyclonal anti-IFI27	abcam	Cat# ab171919
Mouse monoclonal anti-Alpha-Smooth Muscle Actin	Thermo Fisher Scientific	Cat# 14-9760-82; RRID: AB_2572996
Rabbit monoclonal anti-CD20	MedChemExpress	Cat# HY-P86444; RRID: AB_3719158
Rabbit monoclonal anti-CD11c	MedChemExpress	Cat# HY-P83653; RRID: AB_3105755
Rabbit multiclonal anti-CD68	abcam	Cat# ab303565; RRID: AB_3075482
Rabbit polyclonal anti-CD163	MedChemExpress	Cat# HY-P81177; RRID: AB_3103089
Mouse monoclonal anti-CD31	BioLegend	Cat# 303110; RRID: AB_493074

**Biological samples**		

Human muscle biopsy	This study	IRB #2022.366

**Critical commercial assays**		

Visium CytAssist Spatial Gene Expression for FFPE, Human Transcriptome, 6.5 mm, 4 rxns	10× Genomics	PN-1000520
Dual Index Kit TS Set A, 96 rxns	10× Genomics	PN-1000251

**Deposited data**		

Public snRNA-seq data	European Genome-phenome Archive (EGA)	EGA: EGAS50000000310
Raw and processed spatial data	This study, Gene Expression Omnibus (GEO)	GEO: GSE317538

**Software and algorithms**		

Leica Las X office	Leica Microsystems	https://www.leica-microsystems.com/products/microscope-software/p/leica-las-x-ls/
Fiji ImageJ	ImageJ	https://imagej.net/
Space Ranger (v3.0.0)	10× Genomics	https://www.10xgenomics.com
Python (v3.9.19)	https://www.python.org/	https://www.python.org/
Scanpy (v1.10.3)	Wolf et al.^45^	https://github.com/scverse/scanpy
Harmony (v0.1.8)	Korsunsky et al.^8^	https://github.com/immunogenomics/harmony
Cell2location (0.1.4)	Kleshchevnikov et al.^10^	https://github.com/BayraktarLab/cell2location
Scipy-1.13.1	Virtanen et al.^46^	https://docs.scipy.org/doc/scipy-1.13.1/
GSEAPY	Fang et al.^47^	https://github.com/zqfang/GSEApy/
COMMOT (v0.0.3)	Cang et al.^13^	https://github.com/zcang/commot
PROGENy	Badia-i-Mompel et al.^48^	https://github.com/scverse/decoupler
Cell Ranger (v8.0.1)	10× Genomics	https://www.10xgenomics.com
Seurat (v5.0.2)	Satija Lab	https://satijalab.org/seurat
CellChat2	Jin et al.^49^	https://github.com/SiYangming/CellChat2

### Experimental model and study participant details

#### Patient muscle biopsy collection

The study was approved by the Joint Chinese University of Hong Kong-New Territories East Cluster Clinical Research Ethics Committee (Ref. No.: 2022.366). All procedures were performed in accordance with standard operating procedure and the principles of the Declaration of Helsinki and ICH Good Clinical Practice. Informed consent was obtained from all study participants. IIM patients admitted to the rheumatology unit of the Prince of Wales Hospital, Hong Kong, China from 1^st^ May 2023 to 30^th^ June 2024 and fulfilled the 2017 American College of Rheumatology/European League Against Rheumatism (ACR/EULAR)^50^ classification criteria were enrolled. Eleven patients were recruited, comprising 4 clinically amyopathic dermatomyositis (CADM), 2 dermatomyositis (DM), 3 overlap myositis (OM), 1 polymyositis (PM), and 1 immune-mediated necrotizing myopathy (IMNM), aged 42–81 years (median 63 years), including 10 females and 1 male. Conchotome muscle biopsies were collected from patients’ lower limb muscles. Patients’ demographic information, clinical characteristics, and MSA status were documented (Figure S1A and Table S1). Healthy control hamstring muscle samples were collected from three female subjects (aged 70–74 years) during orthopedic surgery.

We acknowledge that sex is not perfectly matched across groups due to limited sample availability. However, this is unlikely to affect our primary conclusions: all three MDA5+ DM patients in the core focus group are female, and the MDA5− comparator group (*n* = 8) contains only one male, minimizing potential sex-related confounding in the MDA5+ versus MDA5− transcriptomic comparison.

### Method details

#### Immunofluorescence

Immunofluorescence was performed according to our prior publications.^51^^,^^52^ Slides were fixed with 4% PFA for 20 min at room temperature and permeabilized in ice-cold methanol for 10 min at −20°C. After 4% BSA blocking for 1 h, the sections were incubated with primary antibodies overnight. The Donkey anti-Mouse IgG (H + L) Highly Cross-Adsorbed Secondary Antibody, Alexa Fluor 488 (Invitrogen, A-21202), and Donkey anti-Rabbit IgG (H + L) Highly Cross-Adsorbed Secondary Antibody, Alexa Fluor 594 (Invitrogen, A-21207) were used as secondary antibodies. Primary antibodies and dilutions were used as follows: CD44 (Abcam, ab316123, 1:100), IFITM3 Rabbit mAb 1:150 (Abclonal, A24337), BST2 Rabbit mAb 1:150 (Abclonal, A23445), SAMHD1 Rabbit mAb (Abclonal, A4607, 1:100), IFI27 1:100 (Abcam, ab171919), α-SMA (Invitrogen, Catalog #14–9760–82, 1:150). Imaging was performed with the Leica DM2500 microsystem. Linear adjustments to brightness and contrast were applied globally and equally across all experimental groups using Leica Las X office software. For immune cell staining, slides were fixed with cold acetone to preserve surface proteins. Primary antibodies and dilutions were used as follows: CD20 (MCE, HY-P86444, 1:150), CD11c (MCE, HY-P83653, 1:150), CD68 (Abcam, ab303565, 1:150), CD163 (MCE, HY-P81177, 1:150), CD31 (BioLegend, 303110, 1:150). Immune cell distance to vessels was measured with Leica Las X office software. The total tissue areas were quantified using Fiji/ImageJ software (version 1.54p).

#### Spatial transcriptomics

Fresh muscle tissues were embedded in Optimal Cutting Temperature (OCT), frozen on dry ice, and stored in −80 °C. Specimens were cryo-sectioned at 10 μm and placed on a blank Fisherbrand Superfrost Plus Microscope Slide. To optimize the run and minimize technical batch variation, samples from two different individuals were placed adjacent to each other on the same slide within a single 6.5 mm × 6.5 mm capture area (The specific pairing of samples per slide/batch is detailed in Table S2). After fixation with chilled MeOH at −20 °C for 30 min, Hematoxylin & Eosin (H&E) staining was performed, following the Visium CytAssist Spatial Gene Expression for Fresh Frozen – Methanol Fixation, H&E Staining, Imaging & Destaining protocol (10× genomics, CG000614-Rev A). Tissue slides were imaged under a microscope, and the ones with the best quality (no bubbles, even H&E staining, no folded/overlapped tissue) were chosen to proceed with destaining using 0.1N HCl. The library preparation followed the user guide of Visium CytAssist Spatial Gene Expression Reagent Kits (10× genomics, CG000495, Rev E). Briefly, after destaining, the tissue slides were incubated with the Human WT Probes v2 overnight (20 h) for hybridization. After ligation, the probes were released and transferred using the Visium CytAssist machine, which facilitates the transfer of transcriptomic probes from standard glass slides to the Visium capturing slides (two 6.5 mm × 6.5 mm capture areas). After extension, probes were eluted from the Visium slides, and pre-amplification of 10 PCR cycles was performed. The final cycle number of the library was determined by quantitative PCR. Libraries were sequenced on the Illumina NovaSeq-6000 system platform, and 25 GB of data were obtained for each capture area.

#### Spatial transcriptomics data processing

After the sequencing was completed, the 10× Genomics Space Ranger (v3.0.0) count function was used to align raw fastq files with the human reference transcriptome (GRCh38-2020-A), tissue detection, fiducial detection and barcode/UMI counting. Subsequent analysis was conducted using Python (v3.9.19). Filtered counts were loaded using scanpy (v1.10.3)^45^ and spots with fewer than 1000 counts, fewer than 200 features, or more than 20% mitochondrial genes were excluded from downstream analysis. After samples were merged, counts per million (CPM) normalization using built-in function sc.pp.normalize_total() and a log transformation using built-in function sc.pp.log1p() were applied to the merged object. sc.pp.highly_variable_genes() function was then used to select highly variable genes with following parameter settings: min_mean = 0.0125, max_mean = 3, min_disp = 0.5. Following the selection of highly variable genes, the sc.pp.scale() function was used to scale the data to zero mean and unit variance. Next, the merged object was subjected to Principal Component Analysis (PCA) using the sc.tl.pca() function with 50 components, and Harmony (v0.1.8)^8^ was used to eliminate the batch effects.

#### Cell type deconvolution

Spatial data was deconvoluted using Cell2location (0.1.4)^10^ with 10 cells per location and a detection α of 20. Unnormalized, single-cell data with cluster information were used as a reference, and q05 abundance counts were used for downstream analysis. Correlations between cell type abundances were calculated using the scipy (v1.13.1)^46^ Pearson’s correlation coefficient. The spatial distribution of multiple cell types was visualized in a unified panel through the built-in plot_spatial() function provided by Cell2location.

#### Niche identification

Non-negative-matrix factorization (NMF) function of the Cell2location package was applied to the q05 estimation of cell type abundance to identify cellular niches. We calculated 2 to 30 factors and found 6 factors to best represent cellular niches. Each of the six resulting cellular niches was assigned a descriptive name based on its distinctive cell type composition.

#### Differentially expressed genes (DEG) and GO analysis

Differentially expressed genes (DEG) analysis was applied to the merged spatial object, which was normalized to counts per million (CPM) and log-transformed using function sc.tl.rank_genes_groups() on each specific group. Unless otherwise specified, differentially expressed genes (DEGs) were defined using the following thresholds: absolute log2 fold change (logFC) > 1.3, adjusted *p*-values (padj) < 0.05, and fraction of cells expressing the gene in the target group (pts) > 0.1. An exception was made for the analyses presented in Figure 1 and its associated Figure S1, where a more stringent logFC cutoff of >2 was applied. GO enrichment analysis was performed on the up-regulated and down-regulated DEGs in each group using built-in function enrichr() provided by GSEAPY (v 1.1.4),^47^ with the gene set ‘GO_Biological_Process_2025.gmt’ as described by Xie et al.^53^

#### Cell-cell communication analysis by COMMOT

For spatial data, COMMOT (v0.0.3)^13^ was used to infer cell–cell communication networks based on ligand–receptor interactions and spatial proximity. Ligand–receptor pairs were obtained from the CellChat database (species = human), restricting to Secreted Signaling interactions. LR pairs were filtered to retain only those where both ligand and receptor genes were expressed in at least 5% of spots (min_cell_pct = 0.05). Spatial communication was then inferred using the built-in function ct.tl.spatial_communication() with a distance threshold of 500 μm (dis_thr = 500) and heteromeric receptor complexes enabled (heteromeric = True). LR pairs were aggregated into signaling pathways (pathway_sum = True), and only pathways containing at least one valid LR pair passing the above filters were retained for downstream analysis, yielding a total of 42 signaling pathways with detectable intercellular communication activity in our dataset.

#### Pathway activity inference by PROGENy

Pathway activities were inferred from the spatially resolved transcriptomic data using the PROGENy method, implemented via the decoupler package (v2.1.1).^48^ The analysis was performed with the function dc.op.progeny(organism = “human”) to generate the pathway model, followed by application of the multivariate linear model dc.mt.ulm() on the preprocessed spatial data. Pathway activity scores for each spot were subsequently extracted using dc.pp.get_obsm (key = “score_ulm”).

#### Functional analysis of immune spots

In the spatial data, spots with the highest 5% abundance for a given immune cell type across all samples were defined as high-abundance spots for that type, collectively referred to as “immune spots.” For instance, spots in the top 5% for MΦ1 were termed “MΦ1 spots.” The same nomenclature was applied to other cell types (e.g., “T cell spots,” “B cell spots”). Following the “immune spots” annotation, DEG analysis was performed to compare the transcriptomes between immune spots (as defined above) and non-immune spots within each niche type. Genes meeting the thresholds of logFC >1.3, adjusted *p*-values (padj) < 0.05, and pts >0.1 were identified as DEGs. These DEGs were then divided into upregulated and downregulated sets for subsequent GO enrichment analysis.

#### snRNA-seq data analysis

The snRNA-seq dataset from a published paper was used, derived from IMNM, IBM, and healthy muscles.^11^ Nuclei fastq files were trimmed and aligned to the human GRCh38-2024-A transcriptome using the 10× Genomics Cell Ranger (v8.0.1) count function.^54^ The R package Seurat (v5.0.2)^55^ was then used to perform the QC, normalization, dimensionality reduction, and clustering on each sample. Nuclei with fewer than 1,000 or more than 20,000 counts, fewer than 800 features, or more than 5% mitochondrial genes were defined as low-quality nuclei and excluded from downstream analysis. Data was normalized using SCTransform^56^ as implemented by Seurat. Subsequently, visualization and clustering were performed using built-in functions in Seurat, including RunUMAP(), FindNeighbors(), and FindClusters(). Next, low-quality clusters were identified using the built-in function FindAllMarkers(), characterized by the absence of marker genes, and the presence of mitochondrial genes or ribosomal genes, and subsequently removed. After excluding all low-quality nuclei, SoupX (v1.6.2)^57^ was employed to eliminate background mRNA contamination. Following the exclusion of all low-quality nuclei and background mRNA contamination, DoubletFinder (v2.0.4)^58^ was used to detect and remove putative doublets on each sample. Subsequently, all samples were integrated, and batch effects were corrected using Seurat CCA algorithm. Finally, normalization, dimensionality reduction and clustering were applied on the integrated object and the built-in function FindAllMarkers() was used to identify the marker genes for each cluster, thus enabling cell-type identification. Importantly, the cell type annotations were fully reproduced from the original reference study. The complete list of marker genes generated from this reproduced analysis and utilized to confirm the cluster annotations is provided in Table S2. For example, the reactive myonuclei (rMN) cluster was identified by high expression of COL19A1, and the stressed myonuclei (sMN) cluster—referred to as ‘damaged myonuclei’ in the original reference—was defined by the expression of the stress-response gene GADD45A. Similarly, T helper (Th) cells were annotated based on high expression of LEF1, IL7R, and THEMIS, whereas cytotoxic T lymphocytes (CTLs) were identified using KLRK1, CCL5, and THEMIS. For a comprehensive list of all annotated cell types and their corresponding marker genes, please refer to the original study.

#### Cell-cell communication analysis of snRNA-seq data

The CellChat2 database (restricted to secreted signaling) and R package^49^ with default parameters were utilized to separately identify biological interactions among different cell types in the same niche using the built-in function aggregateNet(). In this step, all cell types in the same niche were considered as both sources (ligands) and targets (receptors).

### Quantification and statistical analysis

Statistical analyses for non-omics data were performed using GraphPad Prism version 10. Statistical analyses for omics data were performed in Python (version 3.9.19) using SciPy (version 1.13.1). Statistical details for each experiment, including the statistical tests used, exact sample sizes (n), definitions of center and dispersion, and significance levels, are provided in the corresponding figures and figure legends. Unless otherwise specified, differentially expressed genes (DEGs) were defined using the following thresholds: absolute log2 fold change (logFC) > 1.3, adjusted *p*-values (padj) < 0.05, and fraction of cells (spots) expressing the gene in the target group (pts) > 0.1.

### Additional resources

This study did not generate any additional resources.

## Supplemental information

Document S1. Figures S1–S8

Table S1. Clinical information of myositis patients

Table S2. Data quality, DEG, and GO analysis of spatial transcriptomics

Table S3. Quantification of cell-type proportions

Table S4. DEGs of six niche types and proportion of niches across all samples

Table S5. Analysis of DEGs and GO terms among subtypes and serotypes for each niche

Table S6. Analysis of cellular crosstalk and pathway activities

Table S7. DEG and GO analysis of immune cell spots in different niches of MDA5+ muscles
