# Supplementary material for: Spatial transcriptomic mapping of anti-MDA5 dermatomyositis reveals perivascular enrichment of type I interferon responses and immune activation
Source: iScience. 2026 Nov 2;29(11):118072. doi: 10.1016/j.isci.2026.118072 (PMC13755602; doi:10.1016/j.isci.2026.118072)
Supplement: Supplementary file 1 [file mmc1.pdf]

iScience, Volume *29*

Supplemental information

Spatial transcriptomic mapping of anti-MDA5 dermatomyositis reveals perivascular enrichment of type I interferon responses and immune activation

Yulong Qiao, Gexin Liu, Michael Tim-Yun Ong, Hao Sun, Ho So, and Huating Wang

A

Sample ID	Phenotype	Serotype	Age	Gender
Ctrl70	Ctrl	N/A	70	Female
Ctrl73	Ctrl	N/A	73	Female
Ctrl74	Ctrl	N/A	74	Female
CADM42	CADM	anti-MDA5	42	Female
CADM63	CADM	anti-MDA5	63	Female
CADM76	CADM	anti-MDA5	76	Female
CADM47	CADM	anti-Mi2	47	Female
DM70	DM	Nil	70	Male
DM81	DM	anti-TIF1	81	Female
OM49	OM	anti-ro52	49	Female
OM60	OM	anti-ku	60	Female
OM64	OM	Nil	64	Female
PM77	PM	anti-ro52	77	Female
IMNM65	IMNM	anti-HMGCR	65	Female

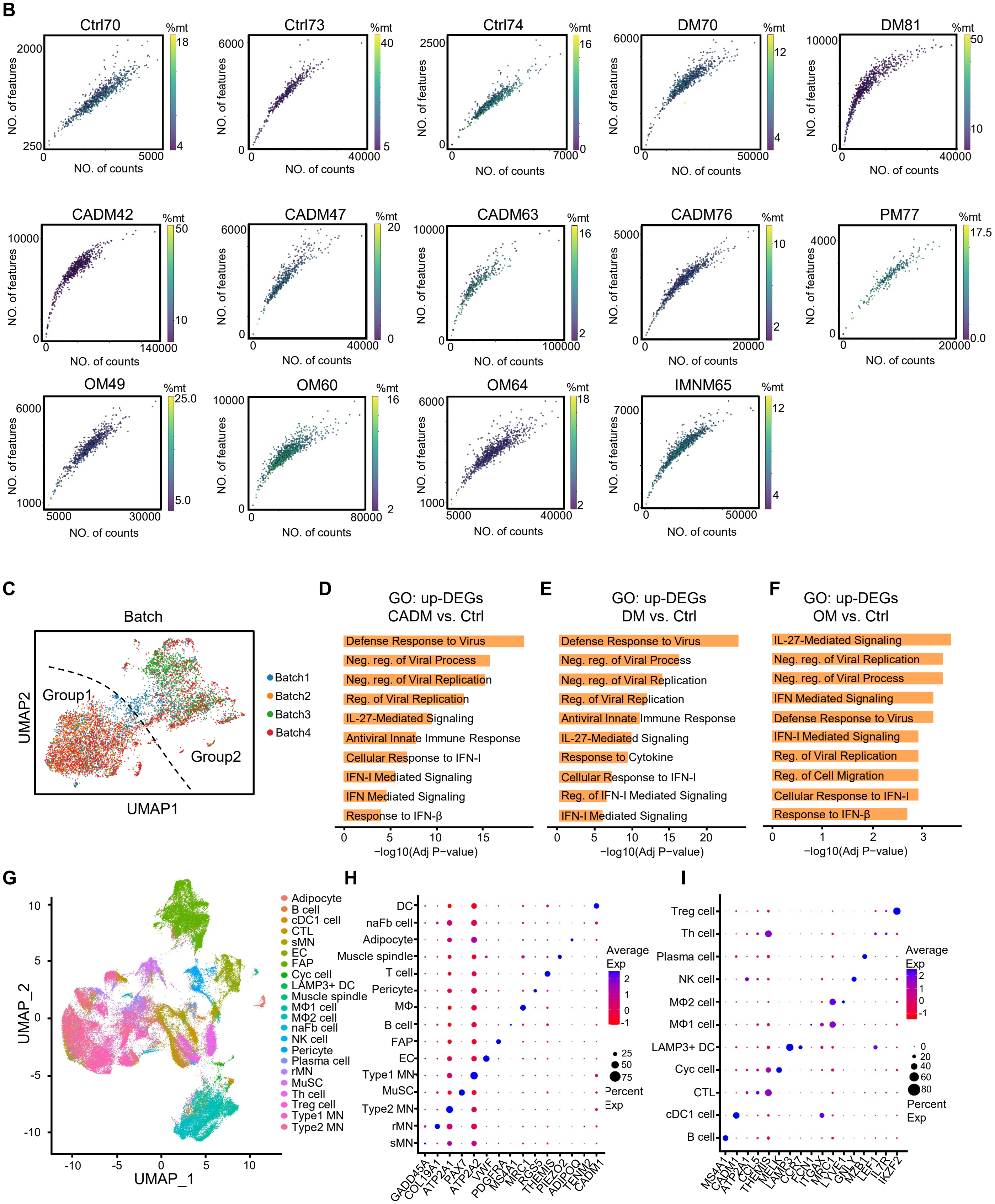

Figure S1. Clinical information and data quality of spatial transcriptomics. (A) Summary of patient clinical characteristics. Detailed information can be found in Supplementary Table S1. (B) Scatter plots showing quality control (QC) metrics: nFeature, nCount, and percentages of mitochondrial encoded genes (%mt) of each sample. (C) UMAP colored by technical batch (slide), demonstrating that the global divergence is driven by biological phenotype rather than technical variation. (D-F) GO plot of biological process (BP) terms enriched in CADM group (D), DM (E), and OM (F) groups using the top 200 upregulated genes, compared to the Ctrl group. (G) UMAP plot showing the 22 cell types identified in the reference snRNA-seq data. (H, I) Dot plot showing marker genes of identified cell types. The annotations were fully reproduced from the reference dataset.

A Estimated cell type abundance in MDA5+, MDA5-, and Ctrl groups

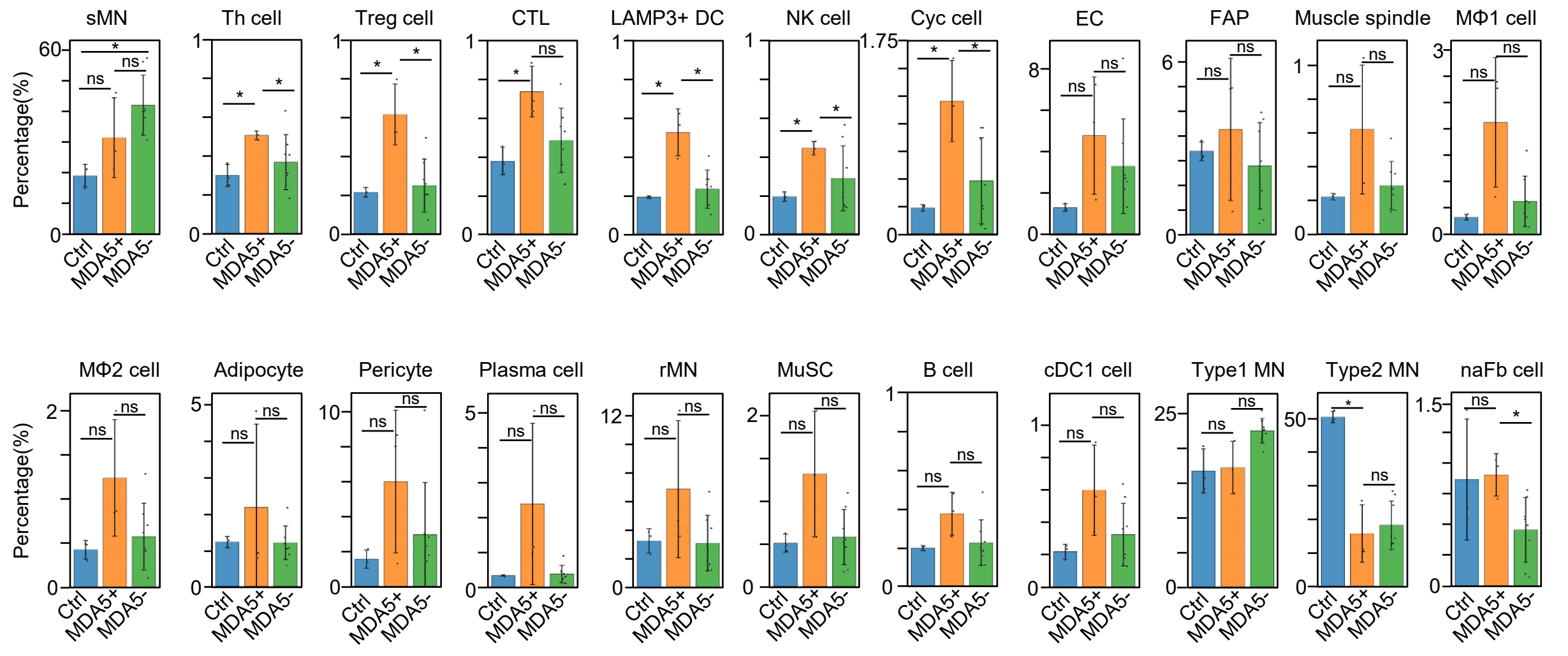

B Cell type abundance in subtypes

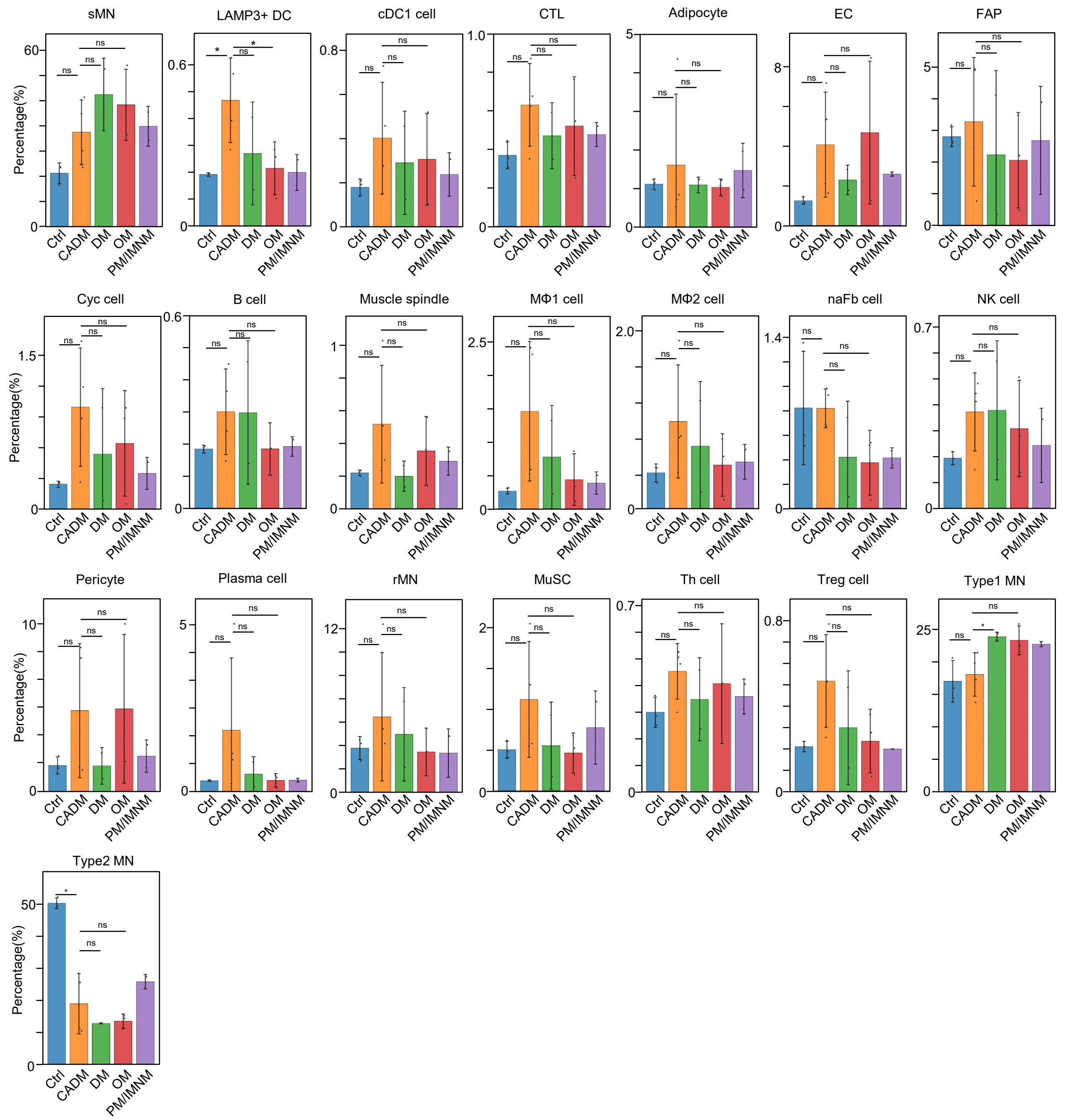

Figure S2. Quantification of cell type abundance. (A) Bar plot showing the percentage of each cell type in Ctrl (n=3), MDA5+ (n=3), and MDA5- groups (n=8). (B) Bar plot showing the percentage of each cell type across myositis subtype: Ctrl (n=3), CADM (n=4), DM (n=2), OM (n=3), and PM/IMNM (n=2). Data are presented as mean ± SD. Statistical significance was determined using an unpaired Welch's t-test (*p < 0.05; ns, not significant).

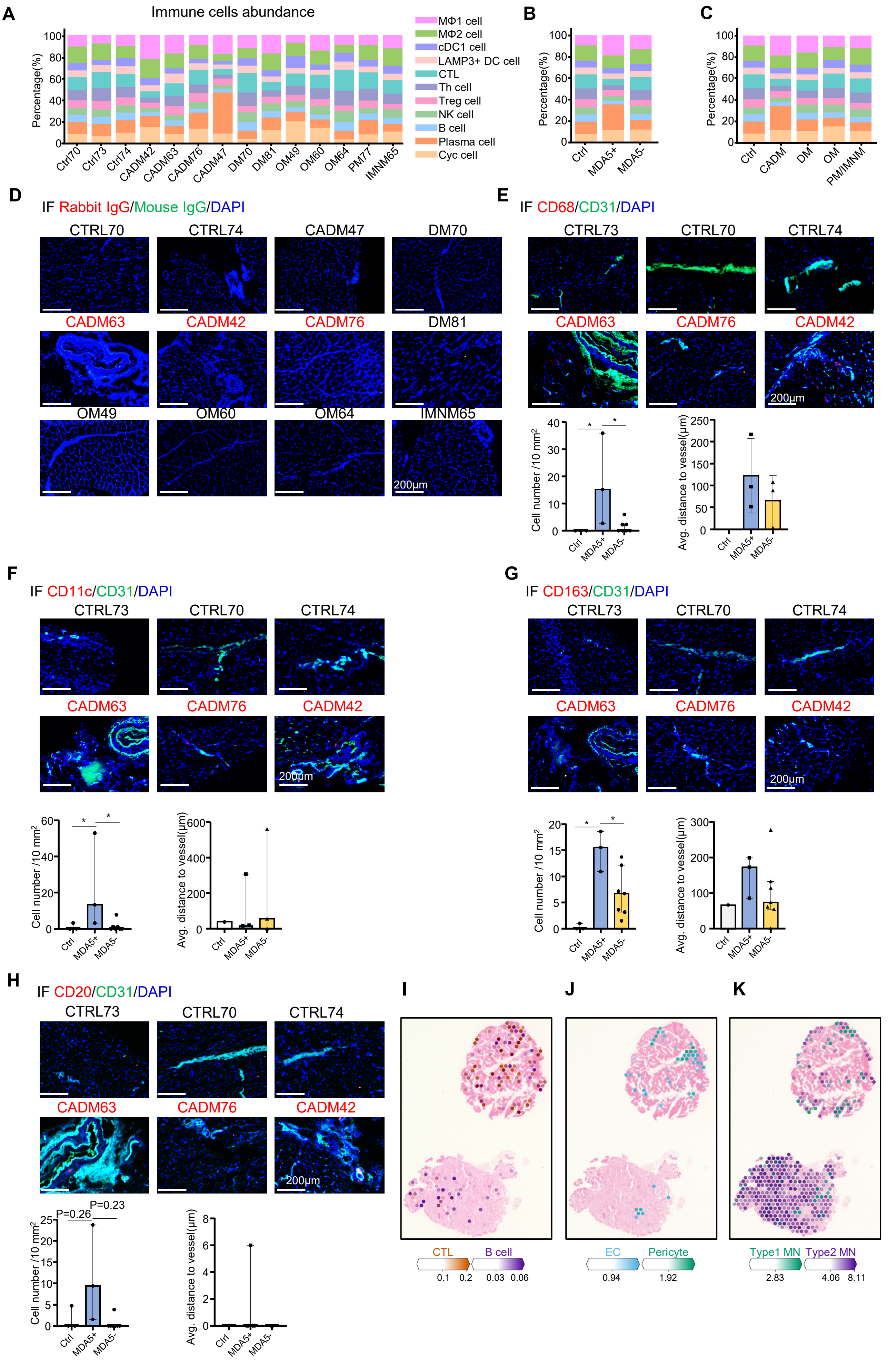
A
Immune cells abundance
Percentage(%)
100
80
60
40
20
0
Ctrl70
Ctrl73
Ctrl74
CADM42
CADM63
CADM76
CADM47
DM70
DM81
OM49
OM60
OM64
PM77
IMNM65
MΦ1 cell
MΦ2 cell
cDC1 cell
LAMP3+ DC cell
CTL
Th cell
Treg cell
NK cell
B cell
Plasma cell
Cyc cell
B
Ctrl
MDA5+
MDA5-
C
Ctrl
CADM
DM
OM
PM/IMNM
D
IF Rabbit IgG/Mouse IgG/DAPI
CTRL70
CTRL74
CADM47
DM70
CADM63
CADM42
CADM76
DM81
OM49
OM60
OM64
IMNM65
200μm
E
IF CD68/CD31/DAPI
CTRL73
CTRL70
CTRL74
CADM63
CADM76
CADM42
200μm
Cell number /10 mm2
Avg. distance to vessel(μm)
F
IF CD11c/CD31/DAPI
CTRL73
CTRL70
CTRL74
CADM63
CADM76
CADM42
200μm
G
IF CD163/CD31/DAPI
CTRL73
CTRL70
CTRL74
CADM63
CADM76
CADM42
200μm
H
IF CD20/CD31/DAPI
CTRL73
CTRL70
CTRL74
CADM63
CADM76
CADM42
200μm
P=0.26
P=0.23
I
J
K
CTL
B cell
0.1
0.2
0.03
0.06
EC
Pericyte
0.94
1.92
Type1 MN
Type2 MN
2.83
4.06
8.11

Figure S3. Quantification and spatial distribution of immune cells. (A) Immune cell composition in each sample, scaled to 100% to reveal the percentage of each cell type. (B) Comparison of immune cell composition in Ctrl, MDA5+, and MDA5- groups. (C) Comparison of immune cell composition in each myositis subtype. (D) Isotype staining of mouse IgG and Rabbit IgG as negative control for immunofluorescence staining. (E-H) Immunofluorescence staining of immune cell markers CD68, CD11c, CD163, CD20. Scale bar = 200 µm. Lower left: Quantification of immune cell numbers per 10 mm^2 section area. Ctrl (n=3), MDA5+ (n=3), MDA5- (n=7). Lower right: Quantification of average distance of immune cells to vessels (µm) (samples without detected immune cells were excluded from distance quantification). Bars represent the mean ± SEM. Statistical significance was determined using an unpaired Welch's t-test (*$p < 0.05$). (I-J) Spatial plots showing the abundance and co-localization of CTL & B cell (I), EC & pericytes (J) in Ctrl73 and CADM63 samples. (K) Spatial plots showing the abundance and separate distribution of Type 1 MNs and Type 2 MNs in Ctrl73 and CADM63 samples.

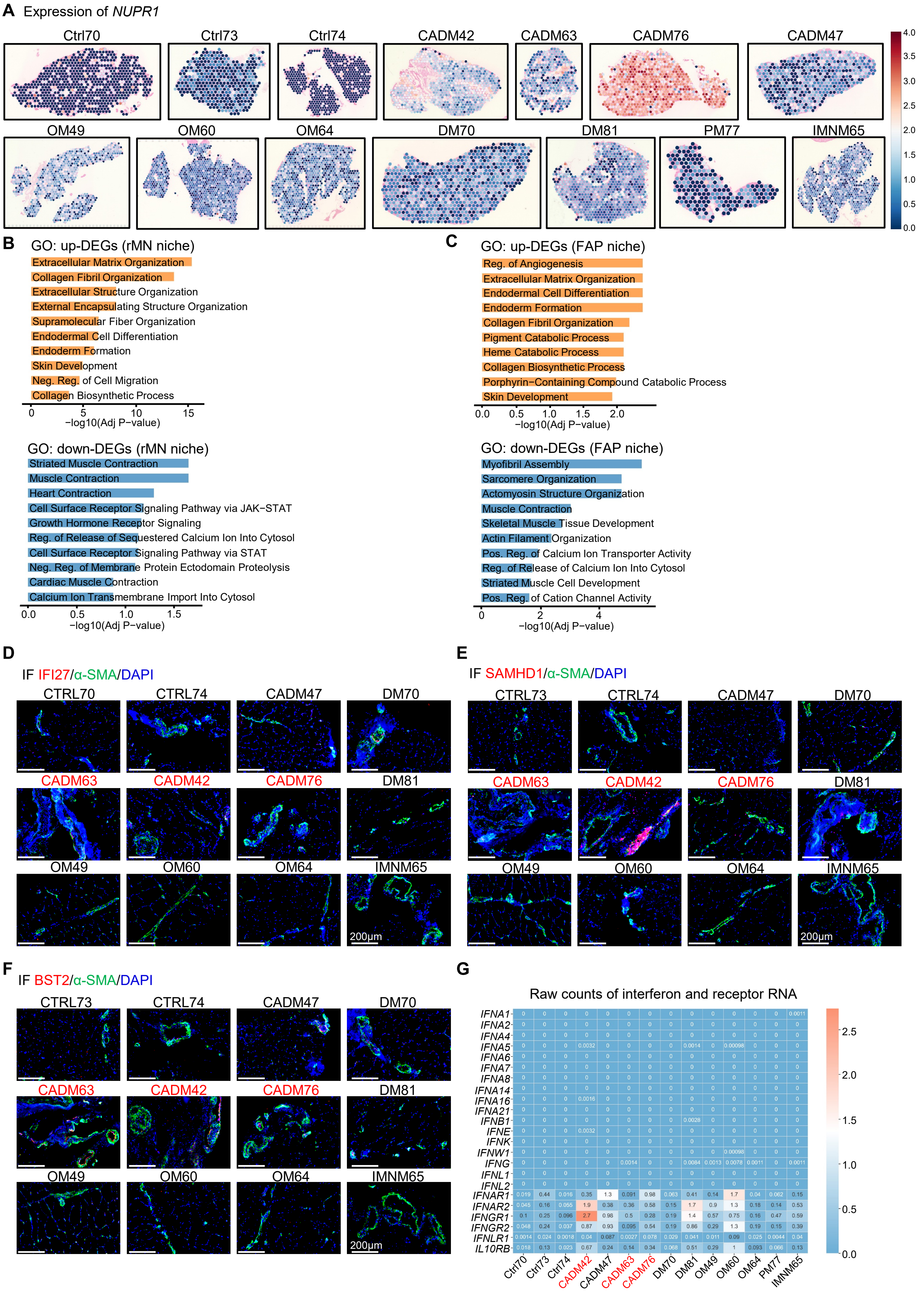
A Expression of NUPR1
Ctrl70
Ctrl73
Ctrl74
CADM42
CADM63
CADM76
CADM47
OM49
OM60
OM64
DM70
DM81
PM77
IMNM65
4.0
3.5
3.0
2.5
2.0
1.5
1.0
0.5
0.0
B
GO: up-DEGs (rMN niche)
Extracellular Matrix Organization
Collagen Fibril Organization
Extracellular Structure Organization
External Encapsulating Structure Organization
Supramolecular Fiber Organization
Endodermal Cell Differentiation
Endoderm Formation
Skin Development
Neg. Reg. of Cell Migration
Collagen Biosynthetic Process
0 5 10 15
−log10(Adj P−value)
GO: down-DEGs (rMN niche)
Striated Muscle Contraction
Muscle Contraction
Heart Contraction
Cell Surface Receptor Signaling Pathway via JAK−STAT
Growth Hormone Receptor Signaling
Reg. of Release of Sequestered Calcium Ion Into Cytosol
Cell Surface Receptor Signaling Pathway via STAT
Neg. Reg. of Membrane Protein Ectodomain Proteolysis
Cardiac Muscle Contraction
Calcium Ion Transmembrane Import Into Cytosol
0.0 0.5 1.0 1.5
−log10(Adj P−value)
C
GO: up-DEGs (FAP niche)
Reg. of Angiogenesis
Extracellular Matrix Organization
Endodermal Cell Differentiation
Endoderm Formation
Collagen Fibril Organization
Pigment Catabolic Process
Heme Catabolic Process
Collagen Biosynthetic Process
Porphyrin−Containing Compound Catabolic Process
Skin Development
0.0 0.5 1.0 1.5 2.0
−log10(Adj P−value)
GO: down-DEGs (FAP niche)
Myofibril Assembly
Sarcomere Organization
Actomyosin Structure Organization
Muscle Contraction
Skeletal Muscle Tissue Development
Actin Filament Organization
Pos. Reg. of Calcium Ion Transporter Activity
Reg. of Release of Calcium Ion Into Cytosol
Striated Muscle Cell Development
Pos. Reg. of Cation Channel Activity
0 2 4
−log10(Adj P−value)
D
IF IFI27/α-SMA/DAPI
CTRL70
CTRL74
CADM47
DM70
CADM63
CADM42
CADM76
DM81
OM49
OM60
OM64
IMNM65
200μm
E
IF SAMHD1/α-SMA/DAPI
CTRL73
CTRL74
CADM47
DM70
CADM63
CADM42
CADM76
DM81
OM49
OM60
OM64
IMNM65
200μm
F
IF BST2/α-SMA/DAPI
CTRL73
CTRL74
CADM47
DM70
CADM63
CADM42
CADM76
DM81
OM49
OM60
OM64
IMNM65
200μm
G
Raw counts of interferon and receptor RNA
IFNA1
IFNA2
IFNA4
IFNA5
IFNA6
IFNA7
IFNA8
IFNA14
IFNA16
IFNA21
IFNB1
IFNE
IFNK
IFNW1
IFNG
IFNL1
IFNL2
IFNAR1
IFNAR2
IFNGR1
IFNGR2
IFNLR1
IL10RB
Ctrl70
Ctrl73
Ctrl74
CADM42
CADM47
CADM63
CADM76
DM70
DM81
OM49
OM60
OM64
PM77
IMNM65
2.5
2.0
1.5
1.0
0.5
0.0

Figure S4. DEG and GO term enrichment in MDA5+ muscle niches. (A) Normalized spatial expression of *NUPR1* in all samples. (B-C) Top 10 enriched GO terms for up- and down-regulated genes in the rMN niche (B) and FAP niche (C). (D-F) Immunofluorescence staining of IFI27 (D), SAMHD1 (E), and BST2 (F) in representative myositis muscles. Nuclei were stained with DAPI (blue). Blood vessels are labeled with α-SMA (green). Scale bar = 200 μm. (G)Heatmap showing the normalized raw counts of the expression of interferon and interferon receptors in each sample.

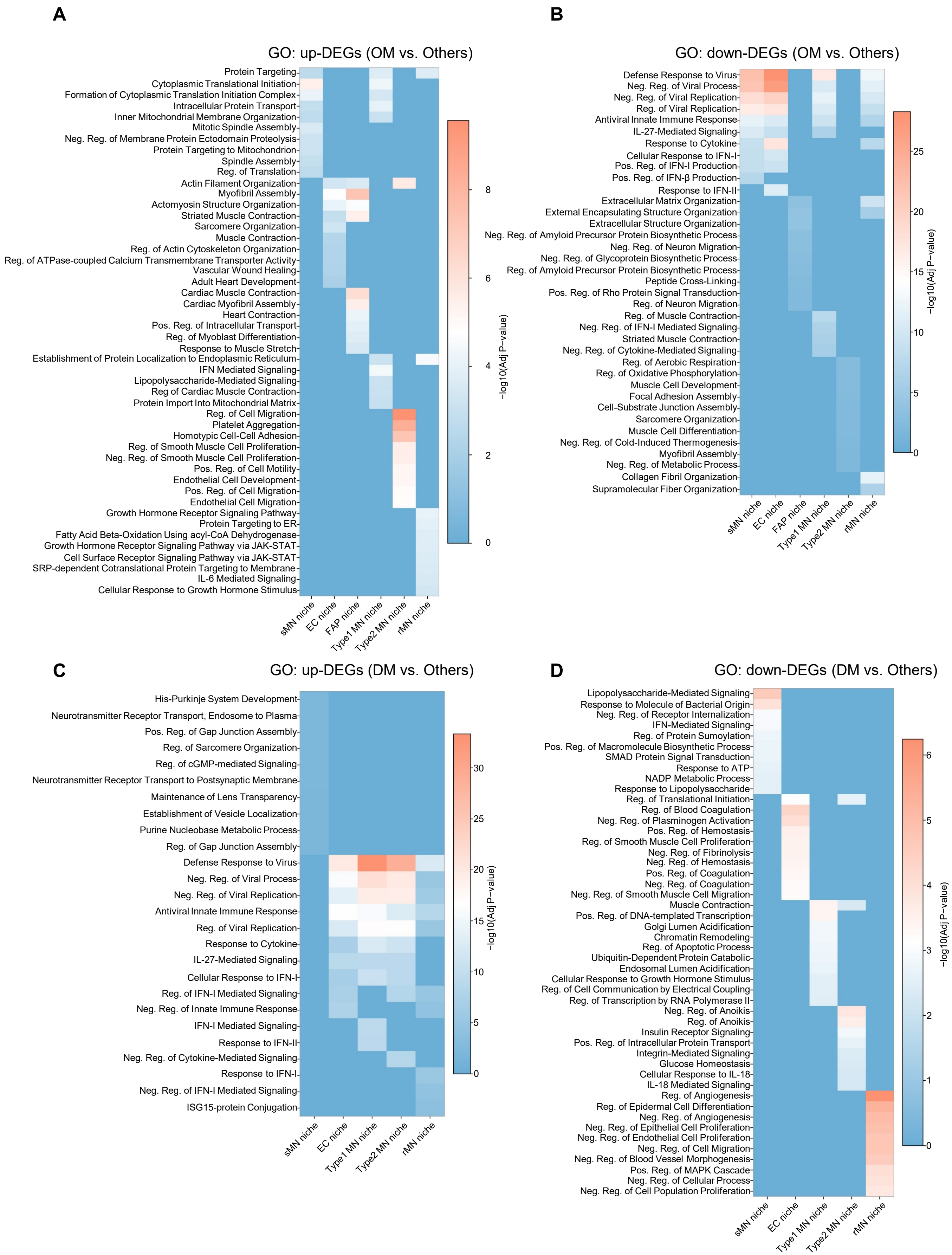

Figure S5. GO analysis of DEGs in OM and DM subtypes. (A-B) Heatmap showing the top 10 enriched GO terms of upregulated genes (A) and down-regulated genes (B) in the six niche types of OM, compared to all the other myositis subtypes, colored by -log10 adjusted P value. (C-D) Heatmap showing the top 10 enriched GO terms of upregulated genes (C) and down-regulated genes (D) in the six niche types of DM (excluding the FAP niche with no GO terms enriched), compared to all the other myositis subtypes, colored by -log10 adjusted P value.

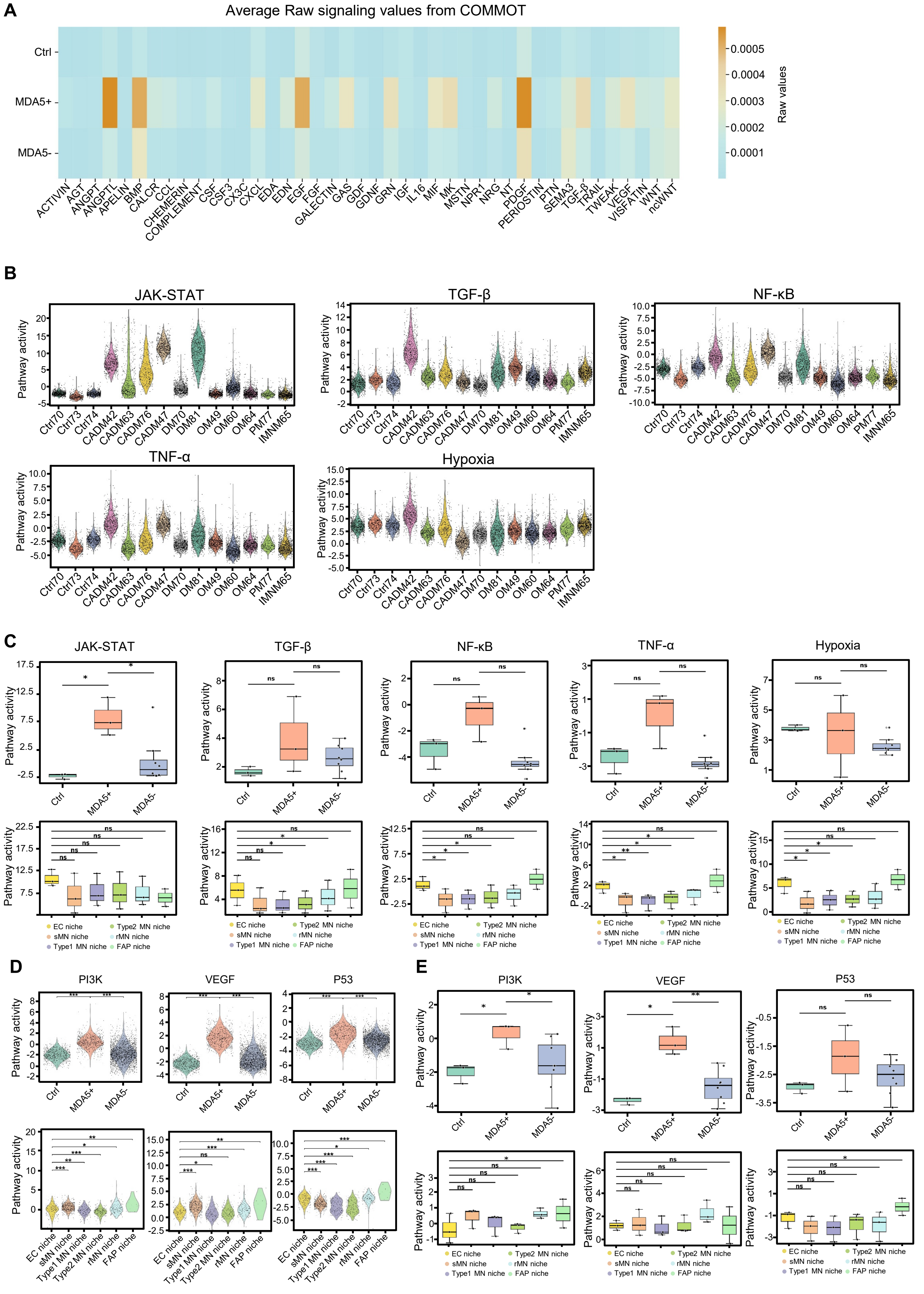
A
Average Raw signaling values from COMMOT
Ctrl
MDA5+
MDA5-
ACTIVIN AGT ANGPT ANGPTL APELIN BMP CALCR CCL CHEMERIN COMPLEMENT CSF CSF3 CX3C CXCL EDA EDN EGF FGF GALECTIN GAS GDF GDNF GRN IGF IL16 MIF MK MSTN NPR1 NRG NT PDGF PERIOSTIN PTN SEMA3 TGF-β TRAIL TWEAK VEGF VISFATIN WNT ncWNT
0.0005
0.0004
0.0003
0.0002
0.0001
Raw values
B
JAK-STAT
TGF-β
NF-κB
TNF-α
Hypoxia
Pathway activity
Ctrl70 Ctrl73 Ctrl74 CADM42 CADM63 CADM76 CADM47 DM70 DM81 OM49 OM60 OM64 PM77 IMNM65
C
JAK-STAT
TGF-β
NF-κB
TNF-α
Hypoxia
Pathway activity
Ctrl
MDA5+
MDA5-
EC niche
sMN niche
Type1 MN niche
Type2 MN niche
rMN niche
FAP niche
D
PI3K
VEGF
P53
Pathway activity
Ctrl
MDA5+
MDA5-
EC niche
sMN niche
Type1 MN niche
Type2 MN niche
rMN niche
FAP niche
E
PI3K
VEGF
P53
Pathway activity
Ctrl
MDA5+
MDA5-
EC niche
sMN niche
Type1 MN niche
Type2 MN niche
rMN niche
FAP niche

Figure S6. Signal communication and pathway activities in myositis muscles. (A) Heatmap showing the normalized signaling communication strength calculated by COMMOT in Ctrl, MDA5+, and MDA5- groups. (B) Violin plots showing the pathway activity of JAK-STAT, Hypoxia, NF-κB, TGF-β, and TNF-α in each sample. (C) Top: Box plot showing the comparison of JAK-STAT, TGF-β, NF-κB, Hypoxia, and TNF-α pathways activity scores among the Ctrl (n=3), MDA5+ (n=3), and MDA5- groups (n=8). Statistical significance was determined using an unpaired Welch's t-test. Bottom: Box plot showing the pathway activity scores across each niche type of MDA5+ muscles. Statistical significance was determined using a paired t-test. Boxes show the median and interquartile range (IQR); whiskers extend to 1.5× IQR. Dots represent individual values. (D) Top: Violin plots showing the pathway activity of PI3K, VEGF, and p53 in the Ctrl, MDA5+, and MDA5- groups at spots level. Bottom: Violin plots showing the pathway activity of PI3K, VEGF, and p53 in the six niches of MDA5+ muscles at spots level. Statistical significance was determined using Mann-Whitney U test (*, p-value < 0.05; **, p-value < 0.01; ***, p-value < 0.001; ns, not significant). (E) Top: Box plots showing the pathway activity of PI3K, VEGF, and p53 in the Ctrl, MDA5+, and MDA5- groups at sample level. Bottom: Box plots showing the pathway activity of PI3K, VEGF, and p53 in the six niches of MDA5+ muscles at sample level.

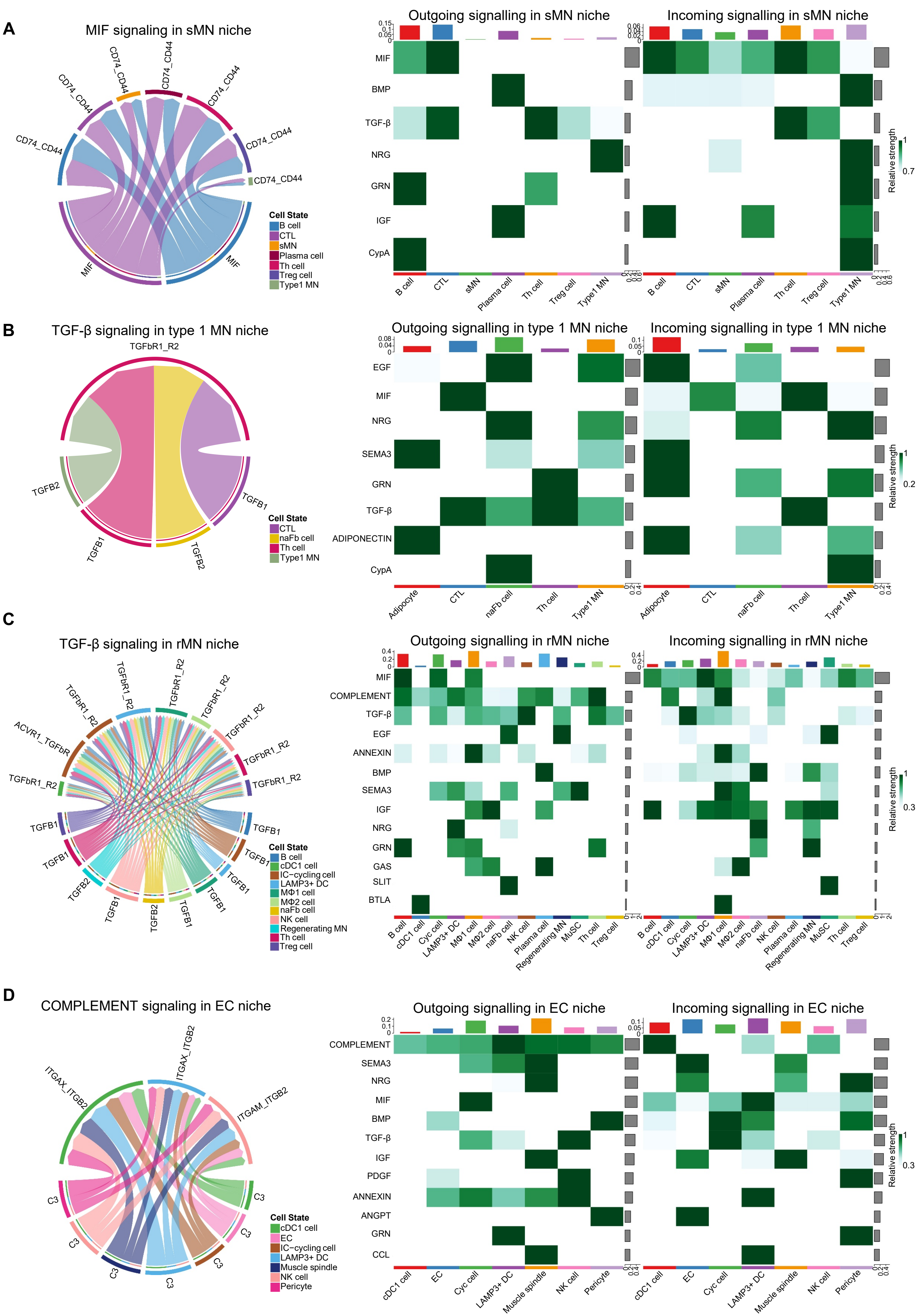

A
MIF signaling in sMN niche
CD74_CD44
MIF
Cell State
B cell
CTL
sMN
Plasma cell
Th cell
Treg cell
Type1 MN
Outgoing signalling in sMN niche
Incoming signalling in sMN niche
MIF
BMP
TGF-β
NRG
GRN
IGF
CypA
B cell
CTL
sMN
Plasma cell
Th cell
Treg cell
Type1 MN
Relative strength
B
TGF-β signaling in type 1 MN niche
TGFbR1_R2
TGFB2
TGFB1
Cell State
CTL
naFb cell
Th cell
Type1 MN
Outgoing signalling in type 1 MN niche
Incoming signalling in type 1 MN niche
EGF
MIF
NRG
SEMA3
GRN
TGF-β
ADIPONECTIN
CypA
Adipocyte
CTL
naFb cell
Th cell
Type1 MN
Relative strength
C
TGF-β signaling in rMN niche
TGFbR1_R2
ACVR1_TGFbR
TGFB1
TGFB2
Cell State
B cell
cDC1 cell
IC−cycling cell
LAMP3+ DC
MΦ1 cell
MΦ2 cell
naFb cell
NK cell
Regenerating MN
Th cell
Treg cell
Outgoing signalling in rMN niche
Incoming signalling in rMN niche
MIF
COMPLEMENT
TGF-β
EGF
ANNEXIN
BMP
SEMA3
IGF
NRG
GRN
GAS
SLIT
BTLA
B cell
cDC1 cell
Cyc cell
LAMP3+ DC
MΦ1 cell
MΦ2 cell
naFb cell
NK cell
Plasma cell
Regenerating MN
MuSC
Th cell
Treg cell
Relative strength
D
COMPLEMENT signaling in EC niche
ITGAX_ITGB2
ITGAM_ITGB2
C3
Cell State
cDC1 cell
EC
IC−cycling cell
LAMP3+ DC
Muscle spindle
NK cell
Pericyte
Outgoing signalling in EC niche
Incoming signalling in EC niche
COMPLEMENT
SEMA3
NRG
MIF
BMP
TGF-β
IGF
PDGF
ANNEXIN
ANGPT
GRN
CCL
cDC1 cell
EC
Cyc cell
LAMP3+ DC
Muscle spindle
NK cell
Pericyte
Relative strength

Figure S7. Cell-cell crosstalk identified from snRNA-seq data. (A-D) Left: Chord diagram visualizing cell-cell communication through MIF signaling in the sMN niche (A), TGF-β signaling in the Type 1 MN niche (B), TGF-β signaling in the rMN niche (C), and COMPLEMENT signaling in the EC niche (D). Right: Heatmaps showing the strength of all the outgoing and incoming signaling between the cell types in the sMN niche (A), Type 1 MN niche (B), rMN niche (C), and EC niche (D).

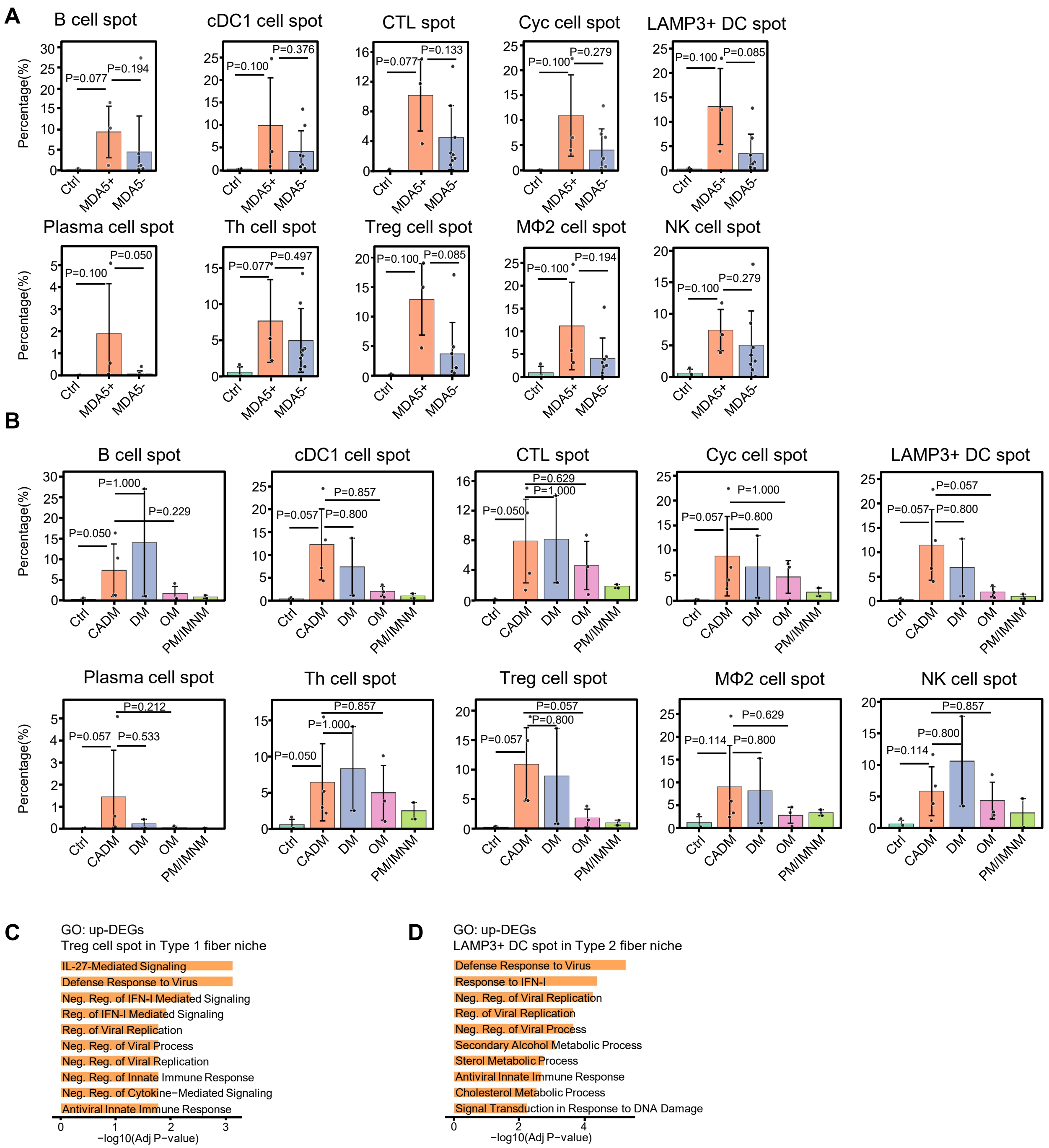

Figure S8. Quantification and analysis of immune cell spots. (A) Bar plots showing the percentage of the 10 types of "immune cell spots" in the Ctrl, MDA5+, and MDA5- groups. Data are presented as mean ± SD, with individual dots representing individual patients. (B) Bar plots showing the percentage of the 10 types of "immune cell spots" in each myositis subtype. Data are presented as mean ± SD, with individual dots representing individual patients. Statistical significance was determined using Mann-Whitney U test. (C) Top 10 enriched GO terms for the upregulated genes of the Treg cell spots in the type 1 MN niche, compared to non-Treg cell spots. (D) Top 10 enriched GO terms for the upregulated genes of the LAMP3+ DC cell spots in type 2 MN niche, compared to non-LAMP3+ DC cell spots.
